# Impact of mesenchymal stem cells’ secretome on glioblastoma pathophysiology

**DOI:** 10.1186/s12967-017-1303-8

**Published:** 2017-10-02

**Authors:** Joana Vieira de Castro, Eduardo D. Gomes, Sara Granja, Sandra I. Anjo, Fátima Baltazar, Bruno Manadas, António J. Salgado, Bruno M. Costa

**Affiliations:** 1 Life and Health Sciences Research Institute (ICVS), School of Medicine, University of Minho, Campus de Gualtar, 4710-057 Braga, Portugal; 2 ICVS/3B’s-PT Government Associate Laboratory, University of Minho, Braga/Guimarães, Campus de Gualtar, 4710-057 Braga, Portugal; 3 CNC-Center for Neuroscience and Cell Biology, University of Coimbra, 3004-517 Coimbra, Portugal; 4 Department of Life Sciences, Faculty of Sciences and Technology, University of Coimbra, 3004-517 Coimbra, Portugal

**Keywords:** Glioblastoma, Mesenchymal stem cells, Human umbilical cord perivascular cells, Conditioned media, Secretome, Viability, Proliferation, Migration, Proteomics

## Abstract

**Background:**

Glioblastoma (GBM) is a highly aggressive primary brain cancer, for which curative therapies are not available. An emerging therapeutic approach suggested to have potential to target malignant gliomas has been based on the use of multipotent mesenchymal stem cells (MSCs), either unmodified or engineered to deliver anticancer therapeutic agents, as these cells present an intrinsic capacity to migrate towards malignant tumors. Nevertheless, it is still controversial whether this innate tropism of MSCs towards the tumor area is associated with cancer promotion or suppression. Considering that one of the major mechanisms by which MSCs interact with and modulate tumor cells is via secreted factors, we studied how the secretome of MSCs modulates critical hallmark features of GBM cells.

**Methods:**

The effect of conditioned media (CM) from human umbilical cord perivascular cells (HUCPVCs, a MSC population present in the Wharton’s jelly of the umbilical cord) on GBM cell viability, migration, proliferation and sensitivity to temozolomide treatment of U251 and SNB-19 GBM cells was evaluated. The in vivo chicken chorioallantoic membrane (CAM) assay was used to evaluate the effect of HUCPVCs CM on tumor growth and angiogenesis. The secretome of HUCPVCs was characterized by proteomic analyses.

**Results:**

We found that both tested GBM cell lines exposed to HUCPVCs CM presented significantly higher cellular viability, proliferation and migration. In contrast, resistance of GBM cells to temozolomide chemotherapy was not significantly affected by HUCPVCs CM. In the in vivo CAM assay, CM from HUCPVCs promoted U251 and SNB-19 tumor cells growth. Proteomic analysis to characterize the secretome of HUCPVCs identified several proteins involved in promotion of cell survival, proliferation and migration, revealing novel putative molecular mediators for the effects observed in GBM cells exposed to HUCPVCs CM.

**Conclusions:**

These findings provide novel insights to better understand the interplay between GBM cells and MSCs, raising awareness to potential safety issues regarding the use of MSCs as stem-cell based therapies for GBM.

**Electronic supplementary material:**

The online version of this article (doi:10.1186/s12967-017-1303-8) contains supplementary material, which is available to authorized users.

## Background

Gliomas are the most common primary malignancies in the central nervous system (CNS), accounting for approximately 80% of all primary brain tumors [1]. Glioblastoma (GBM, grade IV) is the most common and malignant type of glioma in adults, presenting a high mortality rate and very poor patient outcomes. In fact, despite multimodal therapeutic approaches consisting of surgery, chemotherapy and radiotherapy, virtually all GBMs recur and lead to death, presenting a median overall survival of ~ 15 months [2]. This poor outcome has not changed significantly in the last decades, stressing the need for novel therapeutic strategies that may, more efficiently, overcome the highly resistance nature of these tumors. A novel therapeutic approach currently being investigated for a variety of cancer types is based on the use of tumor-trophic stem cells, such as mesenchymal stem cells (MSCs) [3–17]. These are multipotent progenitor cells that are defined according to 3 main characteristics: (i) expression of CD105, CD73 and CD90 (MSCs markers), and lack of expression of CD45, CD34, CD14 (hematopoietic markers); (ii) ability to adhere to plastic surfaces; and (iii) differentiation capacity into adipocytes, osteoblasts and chondrocytes (multipotency) [18, 19]. Additionally, MSCs are also characterized by their proliferative and self-renewal abilities, and can be isolated from bone marrow (BM-MSCs) [20], adipose tissue (ASCs) [21], umbilical cord (e.g., human umbilical cord perivascular cells; HUCPVCs) [22–24], among other sources [25–30]. The use of MSCs is relatively promising since these cells: (i) can be easily isolated and subsequently expanded in vitro; (ii) show multi-lineage differentiation ability; (iii) have an immune privileged nature; (iv) present capacity to home for site of injury, including tumors; and (v) are amenable to genetic modification [31, 32]. In fact, it was already demonstrated that MSCs present an intrinsic capacity to migrate towards gliomas and present low immunogenicity at autologous transplantation [3, 10–12, 33–42]. However, whether this selective MSC tumor-tropism is associated with cancer suppression or promotion functions is still controversial [3, 10, 11, 13, 34, 35, 43]. Several studies, using MSCs engineered to express anti-glioma agents, demonstrated that these cells are highly effective as anti-tumor delivery agents [10, 11, 36, 38, 42, 44, 45]. However, few studies have evaluated the impact of non-engineered MSCs on glioma behavior [3, 12, 13, 46], so it is still unclear if MSCs either promote or repress tumor features. Akimoto and colleagues showed that umbilical cord blood-derived MSCs induced apoptosis in glioma cells; however, in the same study, adipose-derived MSCs enhanced the growth of GBM cells [3]. In another study, co-culturing of adipose-derived MSCs with human glioma cells led to higher survival and proliferation of glioma cells [12], whereas in another study, bone marrow-derived MSCs co-cultured with human glioma cells inhibited tumor cell proliferation [46]. Therefore, further studies focusing on the crosstalk between tumor cells and MSCs should be performed to strengthen the evidence that MSCs-based therapies could be efficiently and safely translated into clinical settings.

This study evaluates how the secretome of a population of MSCs isolated from Wharton Jelly of the umbilical cord (HUCPVCs) modulates critical hallmark features of GBM. In particular, using in vitro and in vivo models, we investigated the effect of HUCPVCs conditioned media (CM) on GBM cells viability, growth, migration, proliferation, angiogenesis, and response to chemotherapy. Proteomic analysis of HUCPVCs CM was performed to identify molecular players that can influence the behavior of GBM cells, which may identify novel targets for therapy.

## Methods

### Cell lines

The human glioblastoma cell line SNB-19 was kindly provided by Rui M. Reis, (Life and Health Sciences Research Institute (ICVS), School of Medicine, University of Minho, Portugal) and the human glioblastoma cell line U251 was kindly provided by Professor Joseph Costello, (Department of Neurological Surgery, University of California, San Francisco (UCSF), USA). Both cell lines were cultured as monolayers in Dulbecco’s Modified Eagle Medium (DMEM; Biochrom) supplemented with 10% (v/v) inactivated fetal bovine serum (FBS; Biochrom), and 1% (v/v) penicillin/streptomycin (Invitrogen). The human umbilical cord perivascular cells (HUCPVCs) were kindly provided by Prof. John E. Davies (University of Toronto, Toronto, Canada), and were previously characterized at the immunophenotype and functional levels [47, 48], and were grown as monolayers in alpha-Minimum Essential Medium (α-MEM; Gibco^®^) supplemented with 10% FBS (Biochrom) and 1% penicillin/streptomycin (Invitrogen). All cell lines were incubated at 37 °C in a humidified atmosphere with 5% (v/v) CO_2_.

### HUCPVCs conditioned media (CM) collection

CM were collected from HUCPVCs in culture at passage 6 (P6). HUCPVCs were plated (at a density of 4 × 10^3^ cells/cm^2^ for in vitro functional assays, or 12 × 10^3^ cells/cm^2^ for proteomic assay) and allowed to grow for 72 h. Subsequently, cells were washed (2 × for in vitro assays, or 5x for proteomic assay) with phosphate buffer solution (PBS) and the medium was replaced by DMEM with 1% penicillin/streptomycin. CM were collected after 48 h (culture media was not renewed or added during this period), filtrated through 0.22 µm filters, immediately snap-frozen and stored at − 80 °C. A total of 7 independent CMs were collected (N = 7), and at least 2 independent CMs were used per each in vitro functional assay, each of which was repeated at least 3 times. For proteomic analysis, 1 CM was used. Before use, CMs were thawed overnight at 4 °C. For the in vitro functional assays, 1% of FBS was added to the media (HUCPVCs CM) as GBM cells are not able to grow without FBS; the control condition consisted of DMEM containing 1% FBS and 1% penicillin/streptomycin (control media). For proteomic analysis, control media consisted of DMEM containing 1% penicillin/streptomycin.

### Cell viability assessment

#### Trypan blue

U251 and SNB-19 GBM cells were plated in triplicates, at an initial density of 5 × 10^4^ and 1 × 10^5^ cells per well in 24-well plates, respectively. After 24 h, HUCPVCs CM or control media were added to cells and incubated for 2, 4, and 6 days (CM and control media were renewed every 48 h). At each time point, total cells were trypsinized and the suspension mixed with trypan blue (1:1 ratio). The number of viable cells were counted in duplicates using hemocytometers. Results represent the mean ± standard deviations (SD) of at least 3 independent experiments.

#### MTT assay

U251 and SNB-19 GBM cells were plated in triplicates, at an initial density of 2.5 × 10^4^ and 5 × 10^4^ cells per well in 48-well plates, respectively. After 24 h, HUCPVCs CM or control media were added to cells and incubated for 2, 4, and 6 days (CM and control media were renewed every 48 h). At each time point, a MTT solution (Thermo Scientific; 0.5 mg of MTT per 1 mL of PBS) was added to each well, followed by incubation in a humidified atmosphere, at 37 °C and 5% (v/v) CO_2_, for 1 h. The optical density was measured at 570 nm using a microplate reader. Results are presented as the mean ± SD of at least 3 independent experiments.

#### Response to temozolomide chemotherapy

To evaluate the effect of HUCPVCs CM on the response of GBM cells to temozolomide (TMZ, Sigma-Aldrich, dissolved in DMSO), the half-maximal inhibitory concentration (IC_50_) of TMZ on U251 and SNB-19 GBM cells was determined by MTT assay. U251 and SNB-19 GBM cells were plated in triplicates, at an initial density of 1 × 10^4^ and 2 × 10^4^ cells per well in 24-well plates, respectively. After 24 h, cells were washed once with PBS and treated with different doses of TMZ (5, 10, 20, 35, 50, 100, 500 μM; or 25, 50, 100, 175, 250, 500, 1000 μM; for U251 and SNB-19 cells, respectively) or vehicle (1% DMSO) in HUCPVCs CM or control media for 5 days (medium with drugs or vehicle was renewed after 2 days). Results are presented as the mean ± SD of 3 independent experiments.

#### Migration (wound healing) assay

U251 and SNB-19 GBM cells were plated in triplicates, at an initial density of 5 × 10^5^ and 2.5 × 10^5^ cells/well in 12-well plates, respectively. After 24 h, a confluent cell monolayer was formed, and a wound was made by manually scratching with a 200 µL pipette tip. Cells in suspension were removed, and adherent cells were washed once with PBS. HUCPVCs CM or control media were carefully added to cells. At this point (0 h), the “wounded” areas were photographed at 4 distinct places, at 40 × magnification by phase contrast microscopy. The same areas were subsequently photographed to monitor wound closure after 16, 24 and 48 h. Migration distances were measured using the be Wound—Cell Migration Tool (Version 1.5) as previously described [49]. Relative wound closure was calculated for each time point. Results are presented as percentages of wound closure and represent the mean ± SD of at least 3 independent experiments.

#### Cell proliferation assay

To evaluate the impact of HUCPVCs CM on GBM cells proliferation, the Cell Proliferation ELISA, 5-bromo-2´-deoxyuridine assay (BrdU, Cell Proliferation ELISA, Applied Sciences) was used as indicated by the manufacturer. Briefly, U251 and SNB-19 GBM cells were plated in triplicates, at an initial density of 1.5 × 10^3^ and 2.5 × 10^3^ cells per well, in 96-well plates, respectively, and grown overnight. Then, adherent cells were treated with HUCPVCs CM or control media for 3 days. After this period, cells were labeled with 10 μL/well of 100 μM BrdU labeling solution, and reincubated for 16 h. BrdU incorporation was assessed according to the manufacturer’s protocol. In order to ensure that the absorbance obtained was a result of proliferating cells, and not simply from a higher number of viable cells, a 96-well plate containing U251 and SNB-19 cells plated and treated in the same conditions as described for BrdU assay, was done to perform MTT assay, following the method described above. Results are presented as the ratio between BrdU positive cells and MTT positive (viable) cells and represent the mean ± SD of 3 independent experiments.

#### Chicken chorioallantoic membrane (CAM) assay

CAM assay was performed as previously described [50]. Briefly, fertilized chicken eggs (supplied by Pinto Bar, Portugal) were incubated at 37 °C in a humidified atmosphere, and on day 3 of development, a window was made into the eggshell after puncturing the air chamber, and eggs were sealed with BTK tape and returned to the incubator. On day 9 of development, 2 × 10^6^ U251 or SNB-19 cells, previously exposed to HUCPVCs CM or control media during 4 days, were re-suspended on 10 µL of Matrigel (BD Biosciences), placed on the CAM, and the eggs were tapped and returned to the incubator. At days 11, 13 and 15 of incubation, 100 µL of new CM or control media was added to each respective group. On developmental day 17, tumors were photographed *in ovo* using a stereomicroscope (Olympus S2x16). The chicken embryos were sacrificed at − 80 °C for 10 min. CAMs and tumors were dissected, fixed in 4% paraformaldehyde at room temperature, and photographed *ex ovo*. The area of the tumors was measured using Cell B software (Olympus), and blood vessels from a selected area containing the tumor were quantified using the Image J software. A total of 38 (U251) and 42 (SNB-19) fertilized chicken eggs were used, 14 (U251) and 15 (SNB-19) in the control group, and 24 (U251) and 27 (SNB-19) in the HUCPVCs CM group.

#### RNA extraction, cDNA synthesis and qRT-PCR

Total RNA from HUCPVCs (N = 3) cultured in the same conditions as for CM collection (P6) was extracted with Trizol (Invitrogen) according to the manufacturer’s instructions. cDNA synthesis was performed using 1 µg of total RNA with High Capacity cDNA Reverse Transcription Kit (Applied Biosystems). Gene-specific mRNA levels were assessed by quantitative real-time PCR (qPCR) in a real-time thermocycler (CFX96; Bio-Rad) using Fast SYBR Green (Qiagen) according to the manufacturer’s instructions, by the 2^−ΔΔCt^ method. The list of primers used can be found in Additional file 1: Table S1.

## Proteomics analysis

### Sample preparation

HUCPVCs CM and control media spiked with the same amount of the recombinant protein *mal*E-GFP (to be use as internal standard) were firstly concentrated using a Vivaspin 20 sample concentrator (5 kDa; GE Healthcare) by centrifugation at 3000 *g*. Concentrated CM and control media were precipitated with Trichloroacetic acid (TCA)-Acetone [51]. The washed pellets were ressuspended in 2 × Laemmli buffer (BioRad)), aided by ultrasonication and denaturated at 95 °C [52]. After denaturation, samples were alkylated with acrylamide and subjected in gel digestion by using the short-GeLC approach [53]. The entire lanes were sliced into 3 parts, and each part was sliced in small pieces and processed. Gel pieces were destained, dehydrated and re-hydrated in 75 µL of trypsin (0.01 µg/µL solution in 10 mM ammonium bicarbonate) for 15 min, on ice. Thirty µL of 10 mM ammonium bicarbonate were then added and in-gel digestion was performed overnight, at room temperature. After digestion, the formed peptides were extracted from the gel pieces by sequential addition of three solutions of acetonitrile (ACN) in 1% formic acid (FA) (30, 50, and 98% of ACN, respectively). All the peptides were dried and subjected to SPE using OMIX tips with C18 stationary phase (Agilent Technologies) as recommended by the manufacture. Eluates were dried and ressuspended with a solution of 2% ACN and 0.1% FA.

### SWATH-MS acquisition

Samples were analyzed on a Triple TOF™ 5600 System (ABSciex^®^) in two different phases: information-dependent acquisition (IDA) and SWATH acquisition. Peptides were resolved by liquid chromatography (nanoLC Ultra 2D, Eksigent^®^) on a MicroLC column ChromXP™ C18CL (300 μm ID × 15 cm length, 3 μm particles, 120 Å pore size, Eksigent^®^) at 5 μL/minutes with a multistep gradient: 0–2 min linear gradient from 5 to 10%, 2–45 min linear gradient from 10 to 30% and, 45–46 min to 35% of ACN in 0.1% FA. Peptides were eluted into the mass spectrometer using an electrospray ionization source (DuoSpray™ Source, ABSciex^®^) with a 50 μm internal diameter (ID) stainless steel emitter (NewObjective). IDA experiments were performed for each 3 peptide mixtures per samples. The mass spectrometer was set to scanning full spectra (350–1250 *m/z*) for 250 ms, followed by up to 100 MS/MS scans (100–1500 *m/z* from a dynamic accumulation time—minimum 30 ms for precursor above the intensity threshold of 1000—with the purpose of maintaining a cycle time of 3.3 s). Candidate ions with a charge state between + 2 and + 5 and counts above a minimum threshold of 10 counts per second were isolated for fragmentation and one MS/MS spectra was collected before adding those ions to the exclusion list for 25 s (mass spectrometer operated by Analyst^®^ TF 1.7, ABSciex^®^). Rolling collision was utilized with a collision energy spread of 5.

The 3 peptide mixtures of each sample were combined and concentrated, and a single analysis of each sample was set for quantitative analysis by acquisition in SWATH mode. For SWATH-MS based experiments, the mass spectrometer was operated in a looped product ion mode [54] and the same chromatographic conditions used as in the IDA run described above. The SWATH-MS setup was specifically designed for the samples to be analyzed (Additional file 2: Table S2), in order to adapt the SWATH windows to the complexity of the set of samples. A set of 60 windows of variable width (containing 1 *m/z* for the window overlap) was conceived covering the precursor mass range of 350–1250 *m/z*. A 250 ms survey scan (350–1500 *m/z*) was acquired at the beginning of each cycle and SWATH MS/MS spectra were collected from 100-1500 m/z for 50 ms resulting in a cycle time of 3.25 s from the precursors ranging from 350 to 1250 *m/z*. The collision energy for each window was determined according to the calculation for a charge +2 ion centered upon the window with variable collision energy spread (CES) according with the window.

A specific library of precursor masses and fragment ions was created by combining all files from the IDA experiments, and used for subsequent SWATH processing. Peptide identification and library generation were performed with Protein Pilot software (v5.1, ABSciex^®^), using the following parameters: (i) search against a database composed by *Homo Sapiens* from SwissProt (release at April 2016), and *malE*-GFP; (ii) acrylamide alkylated cysteines as fixed modification; and (iii) trypsin as digestion type. An independent false discovery rate (FDR) analysis using the target-decoy approach provided with Protein Pilot software was used to assess the quality of the identifications and positive identifications were considered when identified proteins and peptides reached a 5% local FDR [55, 56]. Data processing was performed using SWATH™ processing plug-in for PeakView™ (v2.0.01, ABSciex^®^) as described in [53]. After retention time adjustment using the *mal*E-GFP peptides, up to 15 peptides, with up to 5 fragments each, were chosen per protein, and quantitation was attempted for all proteins in the library file that were identified below 5% local FDR from ProteinPilot™ searches. Peptides’ confidence threshold was determined based on a FDR analysis using the target-decoy approach and the peptides that met the 1% FDR threshold in HUCPVCs sample were retained, and the peak areas of the target fragment ions of those peptides were extracted across the experiments using an extracted-ion chromatogram (XIC) window of 4 min and a XIC width of 100 ppm. The levels of the human proteins were estimated by summing all the filtered transitions from all the filtered peptides for a given protein (an adaptation of [57]) normalized to the internal standard (*malE*-GFP).

### Functional clustering analysis

The identified expressed proteins in HUCPVCs CM were analyzed using the DAVID (Database for Annotation, Visualization and Integrated Discovery) bioinformatics resources version 6.7 (https://david.ncifcrf.gov/) [58, 59]. The list of Uniprot Accession IDs was loaded into the online tool and mapped against reference *Homo sapiens* dataset to extract and summarize functional classification. In DAVID analyses the proteins identified were displayed in Kyoto encyclopedia of genes and genomes (KEGG), Gene ontology (GO), or Reactome pathways.

### Statistical analysis

All statistical analyses were performed using GraphPad Prism 6.0 (GraphPad software, Inc.). To assess the statistical differences between groups, unpaired Student’s *t* test analysis was performed. IC_50_ values were calculated by a nonlinear regression (curve Fit) based on sigmoidal dose-response (variable slope), and two-way repeated-measures analysis of variance (ANOVA) test was used to assess statistical differences between conditions. Results are presented as normalized mean ± SD, and statistical significance was defined as *p* < 0.05 for a 95% confidence interval.

## Results

### HUCPVCs conditioned media (CM) enhance glioblastoma cell viability, migration and proliferation, and do not affect sensitivity to temozolomide chemotherapy

**Fig. 1:**
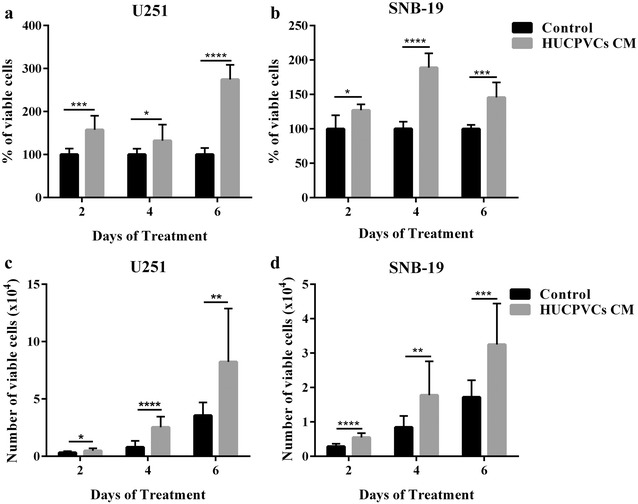
Effect of HUCPVCs conditioned media (CM) on GBM cell viability. Cell viability was measured by MTT (**a**, **b**) and trypan blue (**c**, **d**) assays on U251 (**a**, **c**) and SNB-19 (**b**, **d**) GBM cell lines, after exposure to control media or HUCPVCs CM. HUCPVCs CM led to a statistically significant increase in viability of GBM cells in both assays, in all tested time points. All experiments were done in triplicate, at least in 3 independent experiments. Data is presented as the mean ± SD (*p ≤ 0.05, **p ≤ 0.01, ***p ≤ 0.001 and ****p ≤ 0.0001)

Taking into consideration the controversial reports on the roles of MSCs on tumor behavior, we started by evaluating how the secretome of HUCPVCs modulates critical hallmark features of GBM cells, particularly viability, proliferation and migration. Using two GBM cell lines, U251 and SNB-19, and CM from HUCPVCs, we evaluated GBM cell viability using two complementary assays: MTT (Fig. 1a, b) and trypan blue (Fig. 1c, d). Both U251 and SNB-19 cell lines presented a statistically significant increase in cell viability after exposure to HUCPVCs CM, in all tested time points (shown both by MTT and trypan blue assays; Fig. 1).

**Fig. 2:**
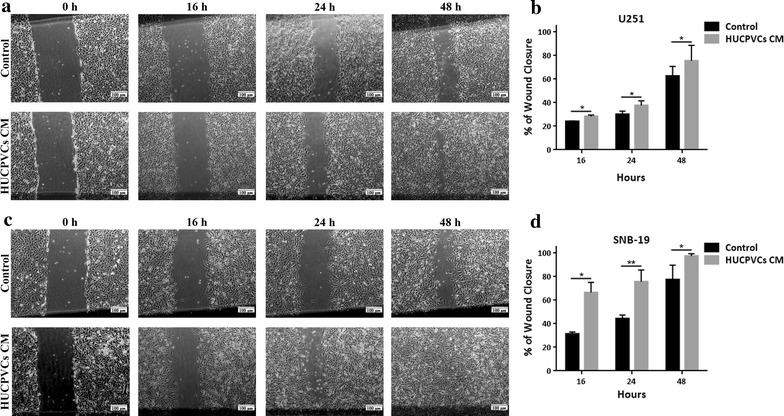
Effect of HUCPVCs conditioned media (CM) on GBM cell migration. **a**, **c** Representative pictures showing the migratory capacity of U251 (**a**) and SNB-19 (**c**) GBM cells exposed to control media or HUCPVCs CM. **b**, **c** Quantification of U251 (**b**) and SNB-19 (**d**) cell migration presented as % of wound closure. Treatment with HUCPVCs CM led to a statistically significant increase of GBM cell migration. Data is presented as the mean ± SD of at least 3 independent experiments, each in triplicate (*p ≤ 0.05 and **p ≤ 0.01)

GBM cell migration was evaluated by a wound healing assay (Fig. 2) on U251 (Fig. 2a, b) and SNB-19 (Fig. 2c, d) cells exposed to HUCPVCs CM. We found that both GBM cell lines, when exposed to CM, presented a statistically significant higher migration capacity when compared to control/unexposed conditions (Fig. 2).

**Fig. 3:**
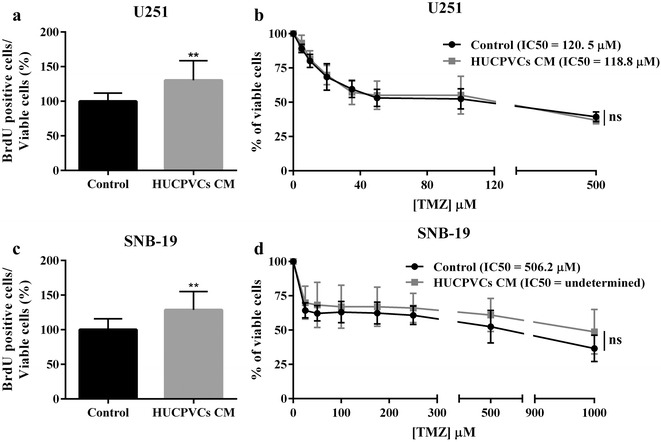
Effect of HUCPVCs conditioned media (CM) on GBM cell proliferation and sensitivity to temozolomide treatment. **a**,** c** Proliferation capacity of U251 (**a**) and SNB-19 (**c**) GBM cells was determined by BrdU assay after treatment with control media or HUCPVCs CM. Exposure to HUCPVCs CM increased the proliferation rate of both GBM cell lines (**p ≤ 0.01). **b**,** d** Determination of the half inhibitory concentration (IC_50_) values of temozolomide (TMZ) treatment in U251 (**b**) and SNB-19 (**d**) cell lines. For both cell lines, no statistically significant differences in the TMZ IC_50_ values were found between cells treated with HUCPVCs CM or control media (*p* = 0.6738 for U251, and *p* = 0.3115 in SNB-19). Results are expressed as the mean ± SD of 3 independent experiments, each in triplicate

Subsequently, the effect of HUCPVCs CM on GBM cell proliferation was evaluated by the BrdU cell proliferation assay (Fig. 3a, c). Both U251 (Fig. 3a) and SNB-19 (Fig. 3c) GBM cells exposed to HUCPVCs CM showed a statistically significant increase in cell proliferation when compared with control conditions.

The influence of HUCPVCs CM exposure on the response of GBM cells to temozolomide (TMZ)-based chemotherapy was then evaluated (Fig. 3b, d). The half inhibitory concentration (IC_50_) values after 5 days of TMZ treatment were determined for U251 (Fig. 3b) and SNB-19 (Fig. 3d) cells. In contrast to the notorious effects previously observed in cell viability, migration, and proliferation, no significant differences were observed in the sensitivity of U251 and SNB-19 cells to TMZ when exposed to HUCPVCs CM versus their respective controls (Fig. 3b, d).

### HUCPVCs conditioned media (CM) increase in vivo tumor growth of GBM cells

**Fig. 4:**
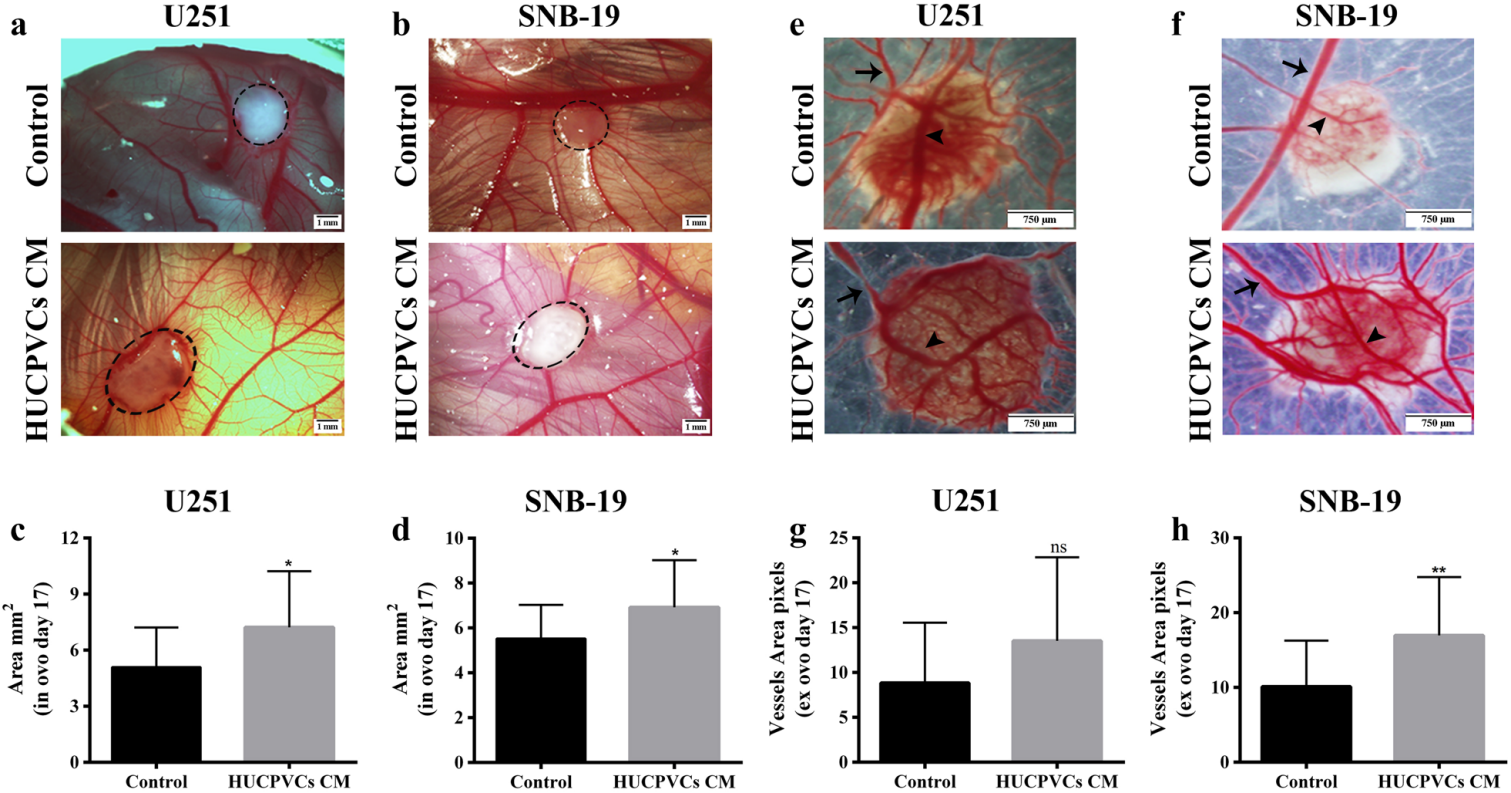
Effect of HUCPVCs conditioned media (CM) in GBM cells growth and angiogenesis, in vivo CAM model. Representative pictures of CAM assay after 8 days of tumor growth *in ovo* (**a**, **b**) and *ex ovo* (**e**, **f**) (× 16 magnification). **c**, **d** Tumor growth was measured *in ovo*. A higher tumor area was found in tumors originated from both U251 (**c**) and SNB-19 (**d**) cells exposed to HUCPVCs CM (*p* = 0.0260 for U251 and *p* = 0.0290 for SNB-19). **g**, **h** Number of blood vessels surrounding tumors derived from U251 (**g**) and SNB-19 (**h**) cells. Both U251 and SNB-19 tumors exposed to HUCPVCs CM presented an increase in the number of blood vessels comparing with control conditions, however only CM-exposed SNB-19 GBM tumors reached statistical significance (*p* = 0.1416 and *p* = 0.0069, respectively). Results are expressed as the mean ± SD (*p ≤ 0.05; **p ≤ 0.01). Dashed circle, tumor; Arrowheads, intra-tumoral blood vessel; Arrow, extra-tumoral blood vessel

In order to complement the in vitro studies, the effect of HUCPVCs CM on GBM 3D tumor growth and angiogenesis was then evaluated using the in vivo Chick Chorioallantoic Membrane (CAM) assay, which allows efficient tumor formation and vascularization [50]. Concordantly with the in vitro data, both U251 (Fig. 4a, c) and SNB-19 (Fig. 4b, d) GBM cells exposed to HUCPVCs CM implanted in the CAM formed statistically significantly larger tumors than those derived from cells exposed to control media (*p* = 0.0260 and *p* = 0.0290, respectively). Regarding vessel density, CM-exposed U251 and SNB-19 GBM tumors presented a general increase in vessel densities compared with control-exposed tumors (Fig. 4e–h), but only SNB-19-derived tumors reached statistical significance (*p* = 0.0069; Fig. 4f, h).

### HUCPVCs conditioned media (CM) contain key proteins involved in cell viability, migration, and proliferation, commonly dysregulated in GBM

Considering the broad consistent effects of HUCPVCs CM on the behavior of tumor cells both in vitro and in vivo, we performed proteomic analyses of HUCPVCs CM to identify the protein content of their secretome that may putatively influence GBM behavior. A total of 699 proteins were identified in our proteomic analysis (N = 1; Additional file 3: Table S3). Concordantly, quantitative RT-qPCR analyses detected mRNA expression of seven genes coding for proteins detected in the proteomic analyses of HUCPVCs (*CCL2*, *TPT1*, *POSTN*, *TGFβI*, *SEMA7A*, *PDGFC*, and *IL6*; Additional file 4: Figure S1), fitting well with our proteomic data.

**Fig. 5:**
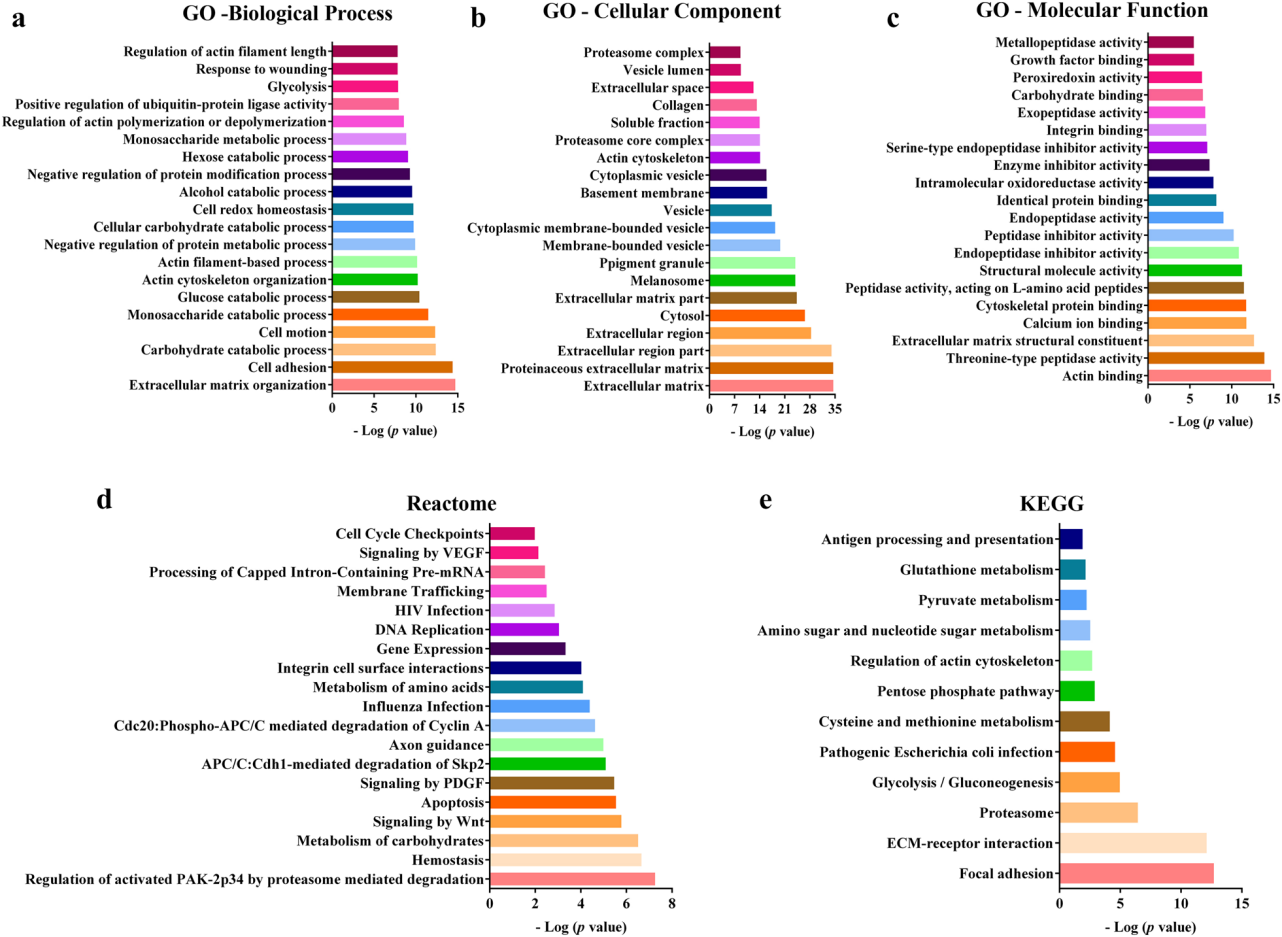
Functional analysis of proteins present in HUCPVCs conditioned media (CM). **a**–**c** DAVID was used to query the functional annotation of HUCPVCs secretome. The top 20 statistically significant enriched Gene Ontology (GO) terms in Biological Process (**a**), Molecular Component (**b**) and Molecular Function (**c**) are shown. **d,** **e** All statistically significant enriched Reactome (**d**) and KEGG (**e**) pathways are represented. The -log values of *p* values are displayed

To better understand the biological functions of these secreted proteins, we employed functional clustering annotation using and integration into Gene Ontology (GO), Kyoto encyclopedia of genes and genomes (KEGG) and Reactome analyses (Fig. 5). Biological processes and cellular components related to extracellular matrix (ECM) organization were the most enriched in HUCPVCs CM (Fig. 5a, b), whereas actin binding was the most represented molecular function among all identified proteins (Fig. 5c). Proteins involved in cell cycle, adhesion, motion, survival, migration, and differentiation, which are well known to be key regulators of a variety of physiological processes but also to be dysregulated in cancer cells, were amongst the most abundantly identified (Fig. 5a). The HUCPVCs secretome was enriched for several pathways by Reactome and KEGG analyses (Fig. 5d, e), including Wnt, platelet-derived growth factor (PDGF), vascular endothelial growth factor (VEGF) and pentose phosphate signaling pathways, as well as proteins involved in focal adhesion, ECM-receptor interaction and DNA replication. Globally, these data identify a set of biological processes and pathways that are well-known to be involved in the regulation of physiological processes, but also to be altered in cancer, which may partly explain the effects of HUCPVCs CM observed in GBM cell behavior.

## Discussion

One of the major concerns in stem cell-based therapies is the impact that modified stem cells may have on tumor behavior. MSCs have been proposed as a new therapeutic approach for glioma treatment, because these cells have been found to have tumor chemotactic capabilities and migrate towards tumor sites through the blood-brain-barrier, where continuous bilateral molecular crosstalks occur between stromal and cancer cells [60, 61]. Although some studies suggested that MSCs inhibit tumor growth, others demonstrated that MSCs have a pro-tumoral function by stimulating tumor growth, migration, invasion, and anticancer-drug resistance [3, 12, 13, 62]. The pro-tumoral effects are mediated by secreted molecules and/or via direct cellular interactions [10, 15, 33–35, 63–70]. Therefore, the clinical validity of MSCs as a potential therapeutic approach for glioma is still a matter of debate, deserving further clarification. In this study, we evaluated the influence of HUCPVCs CM on GBM aggressiveness and highlighted proteins from HUCPVCs CM potentially involved in the observed effects.

Our data shows that GBM cells exposed to HUCPVCs CM exhibit increased viability, migration and proliferation in vitro (Figs. 1, 2, 3a, c). Interestingly, the in vivo CAM model also showed an increase in tumor growth when GBM cells were exposed to HUCPVCs CM (Fig. 4). To the best of our knowledge, this is the first study on the influence of HUCPVCs CM in critical hallmark features of GBM.

Previous studies in different tumor types, including gliomas, are in agreement with our results, showing that MSCs may contribute to tumor growth/proliferation [3, 4, 14, 39]. Additionally, it was also demonstrated that factors released by MSCs increased the migration ability of several types of cancer cells, including breast [71], colon [72], and gastric [73] cancers. Regarding gliomas, Onzi and colleagues demonstrated that ASCs CM treatment was able to increase the migration capacity of U87 GBM cells, which is in line with our results [74]. Interestingly, despite the prominent effects of HUCPVCs CM in multiple dimensions of GBM cell biology, the sensitivity of these tumor cells to TMZ chemotherapy was not significantly affected by HUCPVCs CM (Fig. 3b, d). These results are in agreement with the work of Onzi and colleagues, where they demonstrated that ASCs CM treatment did not alter the response of U87 GBM cells to TMZ [74]. This absence of effect on the response of an anti-tumor drug (doxorubicin) was also recently observed in lung cancer cell lines when exposed to CM from Wharton’s jelly derived MSCs by Hendijani and co-workers [16]. Our study is the first to evaluate the influence of HUCPVCs CM on glioma growth and angiogenesis in a CAM assay with formation of 3D microtumors (Fig. 4). It was previously demonstrated that MSCs can induce angiogenesis in breast and colorectal cancer [75, 76], while in GBM these cells were associated with decreased angiogenesis [46]. In our work, we observed that exposure to HUCPVCs CM increased tumor blood vessels density, particularly in SNB-19 GBM tumors, suggesting that MSCs can induce angiogenesis, which may also contribute to higher tumor growth and tumor aggressiveness. Interestingly, GBM-associated stromal cells (GASCs), which are endogenously present in the tumor microenvironment, were previously shown to present properties of MSCs and have tumor-promoting effects on glioma [77–80], similarly to what we observed with HUCPVCs CM.

Taking into consideration that our study and others showed that MSCs can potentiate tumor aggressiveness, while others demonstrated that MSCs can be safely used as drug delivery agents [10, 39–41], it is crucial to standardize the methods used in different studies to more accurately understand if MSCs are definitely a valid and safe therapeutic approach to tackle cancer. Future studies should have into account several aspects, such as, tissue source and in vitro culture conditions of MSCs; type of tumor cells; variability of experimental methodology; and studies using modified MSCs should include unmodified MSCs as control. In addition, it will also be important to study low-passage primary GBM cell lines, instead of long-term established GBM lines, as these cells resemble more closely the original tumor characteristics, and are thus considered better models.

It is widely accepted that the major mechanism by which MSCs influence cancer pathophysiology is mediated by paracrine events [81–83]. In order to identify which factors secreted by HUCPVCs could be modulating the viability, proliferation, and migration of GBM cells, we performed proteomic analyses of HUCPVCs CM identifying 699 proteins in the secretome. The functional clustering annotation and integration analyses (Fig. 5) revealed that HUCPVCs secretome had a significant enrichment in pathways that have been consistently found dysregulated in cancer (e.g. Wnt, PDGF and VEGF signaling pathways). In fact, these signaling pathways are known to mediate the phenotypes we observed in GBM cells exposed to HUCPVCs CM (namely the increases in proliferation, migration, and invasion), further supporting our experimental findings [84–86]. For example, VEGF is one of the most important regulators of angiogenesis and subsequent tumor growth in GBMs [87–89]. In addition, the expression levels of VEGF in gliomas is correlated with poor prognosis and higher malignancy grades [90, 91]. Regarding PDGF, it was shown that this ligand and its receptors are involved in the proliferation, differentiation and apoptosis of GBM cells, and are commonly overexpressed in GBMs [92, 93]. Finally, overactivation of the Wnt signaling pathway has been associated with several tumor types, including GBM (reviewed in [94]), promoting tumor growth, migration and invasion [95–97]. Additionally, the expression levels of some genes of the Wnt pathway were found to be associated with poor prognosis in glioma patients [97, 98].

Similarly, particular proteins present in the secretome of HUCPVCs (Table 1), such as C-C motif chemokine 2 (CCL2), platelet-derived growth factor C (PDGF-C), semaphorin-7A (Sema-7A), periostin, and interleukin 6 (IL-6) are known to be important regulators of homeostasis in a variety of physiological conditions, but have also been described to influence tumor cell behavior, as is the case of a classic proto-oncogene [99–121]. Interestingly, IL-6, CCL2, and periostin were recently demonstrated to promote M2 macrophage polarization, and thus contribute to tumor growth [122–124].

**Table 1:** Examples of proteins secreted by HUCPVCs that have been described to influence tumor cells’ behavior

Protein (coding gene)	Findings in the context of cancer cells	References
C-C motif chemokine 2 (*CCL2*)	Regulates migration and invasion in several cancer types, including gliomas	[99–101]
Actin-related protein 2/3 complex subunit 5 (*ARPC5*)	Contributes to cell migration and invasion in head and neck squamous cell carcinoma	[102]
Translationally-controlled tumor protein (*TPT1*)	Overexpressed in glioma tissue and is associated with tumor progression and poor clinical outcome of glioma patients. TCTP promotes glioma cell viability and proliferation, in vitro	[103, 104]
Platelet-derived growth factor C (*PDGFC*)	Plays an important role in glioma vessel maturation and stabilization and in the progression of brain tumors, such as glioblastoma and medulloblastoma; and promotes tumor growth by recruitment of cancer-associated fibroblasts	[105–107]
Alpha-actinin-4 (*ACTN4*)	Enhances the motility and invasion potential of various carcinoma cell lines	[108]
Testican-1 (*SPOCK1*)	Promotes the proliferation, migration and invasion and inhibits apoptosis in glioma cells	[109]
Neuropilin-2 (*NRP2*)	Essential for breast cancer tumor initiation being involved in the formation of focal adhesions and is associated with metastasis and poor prognosis; and promotes the invasion and migration of thyroid cancer cells	[110–113]
Disintegrin and metalloproteinase domain-containing protein 10 (*ADAM10*)	Correlated with the grade of malignancy in human glioma; increases the migration capacity of glioma stem cells, and is implicated in U87 cell invasiveness	[114–116]
Transforming growth factor-beta-induced protein (*TGFβI*)	Promotes cell adhesion of human astrocytoma cells, in vitro	[117]
Plasminogen activator inhibitor 1 (*SERPINE1*)	Essential in processes related to tumor development, like angiogenesis, adhesion, migration, invasion and metastasis	[118]
Semaphorin-7A (*SEMA7A*)	Contributes to the increases motility and decreases adhesion necessary for U87 cell invasion	[114]
Periostin (*POSTN*)	Secreted periostin promotes glioma cell invasion and adhesion	[119]
Interleukin 6 (*IL6*)	Secreted IL6 promotes glioma cell invasion and angiogenesis	[120, 121]

## Conclusions

In conclusion, this work shows that HUCPVCs-secreted molecules contribute to GBM aggressiveness, by increasing cell proliferation, migration and viability in vitro, accompanied by higher tumor growth in vivo. The proteomic characterization identifies several putative modulators of these effects in GBM, warranting the need for further studies to understand their mechanisms of action on cancer cells. Our findings also contribute to the understanding of how tumor cells respond to MSCs-released factors, raising concerns about the safety of their use as clinical tools for the treatment of GBM.

## Additional files

**Additional file 1: Table S1.** Sequence of primers used for RT-qPCR analyses.

**Additional file 2: Table S2.** SWATH-MS method.

**Additional file 3: Table S3.** Proteins identified in the secretome of HUCPVCs.

**Additional file 4: Figure S1.** mRNA expression of genes coding for proteins detected in proteomic analyses by quantitative RT-qPCR. (**A**) Gel electrophoresis of *CCL2*, *TPT1*, *POSTN*, *TGFβI*, *SEMA7A*, *PDGFC*, *IL6* and *TBP* expression in HUCPVCs. The RT-qPCR products were run on a 2% agarose gel. (**B**) Relative mRNA expression quantification in HUCPVCs. Data is normalized for *TBP* expression, and results are expressed as the mean ± SD of 3 biological replicates. M, Molecular weight marker 100 bp, ThermoScientific; #1, #2, and #3, independent biological replicates of HUCPVCs; (-), negative control.

## Abbreviations

ACN: acetonitrile
ACTN4: alpha-actinin-4
ADAM10: disintegrin and metalloproteinase domain-containing protein 10
ARPC5: actin-related protein 2/3 complex subunit 5
ASCs: adipose tissue-derived mesenchymal stem cells
BM-MSCs: bone marrow-derived mesenchymal stem cells
CCL2: C–C motif chemokine 2
CES: collision energy spread
CM: conditioned media
CNS: central nervous system
DAVID: database for annotation, visualization and integrated discovery
ECM: extracellular matrix
FA: formic acid
FDR: false discovery rate
GBM: glioblastoma
GO: gene ontology
HUCPVCs: human umbilical cord perivascular cells
IC_50_: half-maximal inhibitory concentration
IDA: information-dependent acquisition
IL-6: interleukin 6
KEGG: Kyoto encyclopedia of genes and genomes
MSCs: mesenchymal stem cells
NRP-2: neuropilin-2
PAI-1: plasminogen activator inhibitor 1
PDGF: platelet-derived growth factor
PDGF-C: platelet-derived growth factor C
SD: standard deviations
Sema7A: semaphorin-7A
TCA: trichloroacetic acid
TCTP: translationally-controlled tumor protein
TGFβIp/ig-h3: transforming growth factor-beta-induced protein/ig-h3
TMZ: temozolomide
VEGF: vascular endothelial growth factor
