# Supplementary material for: Impact of mesenchymal stem cells’ secretome on glioblastoma pathophysiology
Source: J Transl Med. 2017 Oct 2;15:200. doi: 10.1186/s12967-017-1303-8 (PMC5625623; doi:10.1186/s12967-017-1303-8)
Supplement: Supplementary file 1 — Additional file 1: Table S1. Sequence of primers used for RT-qPCR analyses. [file 12967_2017_1303_MOESM1_ESM.docx]

**Table S1:** Sequence of primers used for RT-qPCR analyses.

| **Gene** | **Primer**  **Sense** | **Primer**  **Antisense** | **Primer**  **Tm (°C)** | **Product size (bp)** |
| --- | --- | --- | --- | --- |
| ***CCL2*** | TGTGGTGAGTGTCAACAAGGA | TTGAAGATCTCAAGCGCAGA | 58 | 153 |
| ***TPT1*** | ACTCGCTCATTGGTGGAAAT | TCTTCAAGTTTCCCTTTGATTGA | 55 | 181 |
| ***POSTN*** | CCATGTTTATGGCACTCTGG | CATTCACGTTGCTCTCCAAA | 57 | 179 |
| ***TGFβ1*** | TGTGGTGAGTGTCAACAAGGA | TTGAAGATCTCAAGCGCAGA | 55 | 153 |
| ***SEMA7A*** | AGCTTCGCCTTCAACATCAT | CAGTAGGGGTCTCGGGACAT | 62 | 191 |
| ***PDGFC*** | AAGAAATACGGTCTTGGTATGG | GTTCCATCACTGGGTTCCTC | 57 | 151 |
| ***IL6*** | AAAGAGGCACTGGCAGAAAA | AGCTCTGGCTTGTTCCTCAC | 62 | 183 |
| ***TBP*** | GAGCTGTGATGTGAAGTTTCC | TCTGGGTTTGATCATTCTGTAG | 60 | 118 |

For all genes, qPCR parameters were as follows: 4 minutes at 94 °C, 40 cycles of denaturation for 30 seconds at 94 °C, annealing for 30 seconds, and extension at 72 °C for 30 seconds, and final extension increasing the temperature in 1°C each 5 seconds from 65°C to 95°C.
