# Supplementary material for: Impact of mesenchymal stem cells’ secretome on glioblastoma pathophysiology
Source: J Transl Med. 2017 Oct 2;15:200. doi: 10.1186/s12967-017-1303-8 (PMC5625623; doi:10.1186/s12967-017-1303-8)
Supplement: Supplementary file 2 — Additional file 2: Table S2. SWATH-MS method. [file 12967_2017_1303_MOESM2_ESM.docx]

**Table S2:** SWATH-MS method.

|  | **m/z range** | **Width (Da)** | **CES** |
| --- | --- | --- | --- |
| **Window 1** | 349.5-364.5 | 15 | 5 |
| **Window 2** | 363.5-380.6 | 17.1 | 5 |
| **Window 3** | 379.6-395 | 15.4 | 5 |
| **Window 4** | 394-408.5 | 14.5 | 5 |
| **Window 5** | 407.5-420.7 | 13.2 | 5 |
| **Window 6** | 419.7-432.4 | 12.7 | 5 |
| **Window 7** | 431.4-442.7 | 11.3 | 5 |
| **Window 8** | 441.7-452.2 | 10.5 | 5 |
| **Window 9** | 451.2-461.6 | 10.4 | 5 |
| **Window 10** | 460.6-470.2 | 9.6 | 5 |
| **Window 11** | 469.2-478.7 | 9.5 | 5 |
| **Window 12** | 477.7-487.3 | 9.6 | 5 |
| **Window 13** | 486.3-494.9 | 8.6 | 5 |
| **Window 14** | 493.9-503 | 9.1 | 5 |
| **Window 15** | 502-510.7 | 8.7 | 5 |
| **Window 16** | 509.7-518.8 | 9.1 | 5 |
| **Window 17** | 517.8-526.4 | 8.6 | 5 |
| **Window 18** | 525.4-533.6 | 8.2 | 5 |
| **Window 19** | 532.6-541.3 | 8.7 | 5 |
| **Window 20** | 540.3-549 | 8.7 | 5 |
| **Window 21** | 548-556.2 | 8.2 | 5 |
| **Window 22** | 555.2-564.3 | 9.1 | 5 |
| **Window 23** | 563.3-571.9 | 8.6 | 5 |
| **Window 24** | 570.9-579.1 | 8.2 | 5 |
| **Window 25** | 578.1-586.8 | 8.7 | 5 |
| **Window 26** | 585.8-594.4 | 8.6 | 5 |
| **Window 27** | 593.4-602.5 | 9.1 | 5 |
| **Window 28** | 601.5-610.2 | 8.7 | 5 |
| **Window 29** | 609.2-618.3 | 9.1 | 5 |
| **Window 30** | 617.3-625.9 | 8.6 | 5 |
| **Window 31** | 624.9-634 | 9.1 | 5 |
| **Window 32** | 633-642.6 | 9.6 | 5 |
| **Window 33** | 641.6-651.1 | 9.5 | 5 |
| **Window 34** | 650.1-660.1 | 10 | 5 |
| **Window 35** | 659.1-668.7 | 9.6 | 5 |
| **Window 36** | 667.7-678.1 | 10.4 | 5 |
| **Window 37** | 677.1-687.1 | 10 | 5 |
| **Window 38** | 686.1-697 | 10.9 | 5 |
| **Window 39** | 696-706.9 | 10.9 | 5 |
| **Window 40** | 705.9-716.8 | 10.9 | 5 |
| **Window 41** | 715.8-727.2 | 11.4 | 5 |
| **Window 42** | 726.2-738 | 11.8 | 5 |
| **Window 43** | 737-748.8 | 11.8 | 5 |
| **Window 44** | 747.8-760.5 | 12.7 | 5 |
| **Window 45** | 759.5-772.6 | 13.1 | 5 |
| **Window 46** | 771.6-785.7 | 14.1 | 5 |
| **Window 47** | 784.7-799.2 | 14.5 | 5 |
| **Window 48** | 798.2-813.6 | 15.4 | 8 |
| **Window 49** | 812.6-829.3 | 16.7 | 8 |
| **Window 50** | 828.3-845.1 | 16.8 | 8 |
| **Window 51** | 844.1-861.3 | 17.2 | 8 |
| **Window 52** | 860.3-878.4 | 18.1 | 8 |
| **Window 53** | 877.4-897.7 | 20.3 | 8 |
| **Window 54** | 896.7-920.2 | 23.5 | 8 |
| **Window 55** | 919.2-949.9 | 30.7 | 8 |
| **Window 56** | 948.9-984.1 | 35.2 | 8 |
| **Window 57** | 983.1-1020.1 | 37 | 10 |
| **Window 58** | 1019.1-1064.2 | 45.1 | 10 |
| **Window 59** | 1063.2-1135.3 | 72.1 | 10 |
| **Window 60** | 1134.3-1249.6 | 115.3 | 10 |
