# Supplementary material for: Impact of mesenchymal stem cells’ secretome on glioblastoma pathophysiology
Source: J Transl Med. 2017 Oct 2;15:200. doi: 10.1186/s12967-017-1303-8 (PMC5625623; doi:10.1186/s12967-017-1303-8)
Supplement: Supplementary file 3 — Additional file 3: Table S3. Proteins identified in the secretome of HUCPVCs. [file 12967_2017_1303_MOESM3_ESM.docx]

**Table S3**. Proteins identified in the secretome of HUCPVCs.

| **No. Identified Peptides** | **No. Quantified Peptides** | **Uniprot Accesion Name** | **Protein Name** | **Relative Protein Levels (normalized to internal standard)** | | **Ratio HUCPVCs/Ctrl** |
| --- | --- | --- | --- | --- | --- | --- |
|  |  |  |  |  |  |  |
|  |  |  |  | **Control** | **HUCPVCs** |  |
| 4 | 1 | CCL2_HUMAN | C-C motif chemokine 2 | 0.000 | 0.036 | 709.1 |
| 1 | 1 | MOXD1_HUMAN | DBH-like monooxygenase protein 1 | 0.000 | 0.009 | 221.9 |
| 4 | 1 | ARPC5_HUMAN | Actin-related protein 2/3 complex subunit 5 | 0.000 | 0.008 | 154.0 |
| 1 | 1 | PALLD_HUMAN | Palladin | 0.000 | 0.004 | 104.6 |
| 8 | 5 | TCTP_HUMAN | Translationally-controlled tumor protein | 0.000 | 0.045 | 92.2 |
| 1 | 1 | LYOX_HUMAN | Protein-lysine 6-oxidase | 0.000 | 0.002 | 87.2 |
| 54 | 15 | MMP2_HUMAN | 72 kDa type IV collagenase | 0.017 | 1.421 | 84.3 |
| 1 | 1 | PDGFC_HUMAN | Platelet-derived growth factor C | 0.000 | 0.002 | 77.6 |
| 61 | 15 | PXDN_HUMAN | Peroxidasin homolog | 0.003 | 0.219 | 72.4 |
| 32 | 14 | PDIA3_HUMAN | Protein disulfide-isomerase A3 | 0.004 | 0.266 | 70.4 |
| 59 | 14 | ACTN4_HUMAN | Alpha-actinin-4 {ECO:0000305} | 0.003 | 0.220 | 67.8 |
| 8 | 6 | TICN1_HUMAN | Testican-1 | 0.001 | 0.045 | 67.4 |
| 2 | 1 | ADHX_HUMAN | Alcohol dehydrogenase class-3 | 0.000 | 0.006 | 66.5 |
| 4 | 2 | PTPA_HUMAN | Serine/threonine-protein phosphatase 2A activator | 0.000 | 0.010 | 63.4 |
| 39 | 14 | QSOX1_HUMAN | Sulfhydryl oxidase 1 | 0.007 | 0.412 | 63.1 |
| 1 | 1 | STIP1_HUMAN | Stress-induced-phosphoprotein 1 | 0.000 | 0.002 | 62.6 |
| 33 | 14 | C1R_HUMAN | Complement C1r subcomponent | 0.005 | 0.294 | 62.4 |
| 26 | 11 | CO5A1_HUMAN | Collagen alpha-1(V) chain | 0.002 | 0.139 | 62.2 |
| 2 | 1 | EPCR_HUMAN | Endothelial protein C receptor | 0.000 | 0.007 | 61.8 |
| 18 | 13 | PGS1_HUMAN | Biglycan | 0.007 | 0.422 | 60.6 |
| 13 | 8 | TXND5_HUMAN | Thioredoxin domain-containing protein 5 | 0.001 | 0.064 | 60.3 |
| 1 | 1 | SEC13_HUMAN | Protein SEC13 homolog | 0.000 | 0.002 | 60.3 |
| 1 | 1 | NIF3L_HUMAN | NIF3-like protein 1 {ECO:0000305\|PubMed:11124544} | 0.000 | 0.003 | 60.0 |
| 42 | 15 | NID2_HUMAN | Nidogen-2 | 0.003 | 0.182 | 60.0 |
| 3 | 2 | BTD_HUMAN | Biotinidase | 0.000 | 0.014 | 59.2 |
| 2 | 2 | CO8A1_HUMAN | Collagen alpha-1(VIII) chain | 0.000 | 0.008 | 57.9 |
| 21 | 15 | PTX3_HUMAN | Pentraxin-related protein PTX3 | 0.024 | 1.346 | 56.8 |
| 33 | 14 | NRP2_HUMAN | Neuropilin-2 | 0.002 | 0.128 | 56.1 |
| 37 | 15 | CSTN1_HUMAN | Calsyntenin-1 | 0.012 | 0.660 | 55.5 |
| 22 | 14 | GDN_HUMAN | Glia-derived nexin | 0.006 | 0.312 | 55.5 |
| 95 | 14 | FLNA_HUMAN | Filamin-A | 0.005 | 0.299 | 55.2 |
| 3 | 2 | NUCB2_HUMAN | Nucleobindin-2 | 0.000 | 0.007 | 54.3 |
| 6 | 3 | ADA10_HUMAN | Disintegrin and metalloproteinase domain-containing protein 10 | 0.000 | 0.016 | 54.0 |
| 1 | 1 | STRAP_HUMAN | Serine-threonine kinase receptor-associated protein | 0.000 | 0.001 | 53.9 |
| 2 | 1 | S10A6_HUMAN | Protein S100-A6 | 0.000 | 0.005 | 53.8 |
| 13 | 11 | PNPH_HUMAN | Purine nucleoside phosphorylase | 0.002 | 0.088 | 52.4 |
| 37 | 15 | ACTN1_HUMAN | Alpha-actinin-1 | 0.006 | 0.319 | 52.3 |
| 33 | 15 | TSP2_HUMAN | Thrombospondin-2 | 0.006 | 0.322 | 52.0 |
| 8 | 3 | STC2_HUMAN | Stanniocalcin-2 | 0.002 | 0.102 | 50.1 |
| 2 | 1 | FBLN5_HUMAN | Fibulin-5 | 0.000 | 0.008 | 49.3 |
| 30 | 15 | PLST_HUMAN | Plastin-3 | 0.005 | 0.245 | 48.6 |
| 47 | 16 | BGH3_HUMAN | Transforming growth factor-beta-induced protein ig-h3 | 0.044 | 2.137 | 48.3 |
| 8 | 7 | CTHR1_HUMAN | Collagen triple helix repeat-containing protein 1 | 0.003 | 0.151 | 48.3 |
| 108 | 15 | CO6A3_HUMAN | Collagen alpha-3(VI) chain | 0.004 | 0.214 | 47.9 |
| 4 | 2 | IF4A2_HUMAN | Eukaryotic initiation factor 4A-II | 0.000 | 0.010 | 47.8 |
| 29 | 13 | CO5A2_HUMAN | Collagen alpha-2(V) chain | 0.003 | 0.126 | 47.6 |
| 2 | 1 | ARP2_HUMAN | Actin-related protein 2 | 0.000 | 0.006 | 47.4 |
| 11 | 5 | PEPD_HUMAN | Xaa-Pro dipeptidase | 0.001 | 0.030 | 47.3 |
| 4 | 3 | RBMX_HUMAN | RNA-binding motif protein, X chromosome | 0.000 | 0.018 | 47.2 |
| 50 | 15 | TSP1_HUMAN | Thrombospondin-1 | 0.012 | 0.576 | 47.1 |
| 58 | 15 | CO6A1_HUMAN | Collagen alpha-1(VI) chain | 0.035 | 1.626 | 46.5 |
| 37 | 15 | PAI1_HUMAN | Plasminogen activator inhibitor 1 | 0.060 | 2.769 | 46.1 |
| 8 | 3 | PSB4_HUMAN | Proteasome subunit beta type-4 | 0.001 | 0.031 | 46.1 |
| 20 | 14 | CATB_HUMAN | Cathepsin B | 0.007 | 0.306 | 45.8 |
| 31 | 15 | MANBA_HUMAN | Beta-mannosidase | 0.004 | 0.193 | 45.7 |
| 40 | 15 | MMP1_HUMAN | Interstitial collagenase | 0.037 | 1.683 | 45.7 |
| 37 | 15 | CO4A2_HUMAN | Collagen alpha-2(IV) chain | 0.007 | 0.325 | 45.7 |
| 100 | 15 | FBN1_HUMAN | Fibrillin-1 | 0.009 | 0.392 | 45.5 |
| 29 | 15 | SEM7A_HUMAN | Semaphorin-7A | 0.005 | 0.209 | 44.8 |
| 22 | 14 | FBLN1_HUMAN | Fibulin-1 | 0.003 | 0.143 | 44.6 |
| 1 | 1 | MINP1_HUMAN | Multiple inositol polyphosphate phosphatase 1 | 0.000 | 0.006 | 44.4 |
| 44 | 14 | MOES_HUMAN | Moesin | 0.008 | 0.356 | 44.1 |
| 3 | 1 | CALD1_HUMAN | Caldesmon | 0.000 | 0.003 | 43.8 |
| 96 | 14 | CO3_HUMAN | Complement C3 | 0.008 | 0.367 | 43.7 |
| 4 | 2 | CPPED_HUMAN | Serine/threonine-protein phosphatase CPPED1 | 0.000 | 0.005 | 43.5 |
| 20 | 12 | PGS2_HUMAN | Decorin | 0.004 | 0.186 | 43.4 |
| 2 | 1 | ENOPH_HUMAN | Enolase-phosphatase E1 {ECO:0000255\|HAMAP-Rule:MF_03117} | 0.000 | 0.004 | 42.8 |
| 1 | 1 | SC23A_HUMAN | Protein transport protein Sec23A | 0.000 | 0.002 | 42.8 |
| 9 | 8 | VASN_HUMAN | Vasorin | 0.002 | 0.090 | 42.6 |
| 2 | 1 | BACH_HUMAN | Cytosolic acyl coenzyme A thioester hydrolase | 0.000 | 0.004 | 42.4 |
| 23 | 15 | ECM1_HUMAN | Extracellular matrix protein 1 | 0.007 | 0.307 | 42.3 |
| 142 | 15 | FINC_HUMAN | Fibronectin | 0.045 | 1.887 | 42.3 |
| 2 | 2 | PGFRL_HUMAN | Platelet-derived growth factor receptor-like protein | 0.000 | 0.003 | 42.0 |
| 2 | 1 | OTUB1_HUMAN | Ubiquitin thioesterase OTUB1 | 0.000 | 0.003 | 41.9 |
| 87 | 16 | CO1A2_HUMAN | Collagen alpha-2(I) chain | 0.036 | 1.513 | 41.8 |
| 7 | 2 | KAD2_HUMAN | Adenylate kinase 2, mitochondrial {ECO:0000255\|HAMAP-Rule:MF_03168} | 0.000 | 0.009 | 41.8 |
| 3 | 3 | EXTL2_HUMAN | Exostosin-like 2 | 0.000 | 0.009 | 41.6 |
| 39 | 12 | LAMB1_HUMAN | Laminin subunit beta-1 | 0.003 | 0.111 | 41.6 |
| 11 | 5 | TIMP1_HUMAN | Metalloproteinase inhibitor 1 | 0.007 | 0.276 | 41.5 |
| 7 | 6 | LMAN2_HUMAN | Vesicular integral-membrane protein VIP36 | 0.001 | 0.024 | 41.3 |
| 9 | 2 | PRDX5_HUMAN | Peroxiredoxin-5, mitochondrial | 0.000 | 0.013 | 41.2 |
| 4 | 2 | DX39B_HUMAN | Spliceosome RNA helicase DDX39B | 0.000 | 0.008 | 41.1 |
| 17 | 14 | OLFL3_HUMAN | Olfactomedin-like protein 3 | 0.007 | 0.278 | 41.1 |
| 46 | 15 | LTBP1_HUMAN | Latent-transforming growth factor beta-binding protein 1 | 0.003 | 0.127 | 40.9 |
| 12 | 10 | GAS6_HUMAN | Growth arrest-specific protein 6 | 0.001 | 0.060 | 40.9 |
| 3 | 2 | DOPD_HUMAN | D-dopachrome decarboxylase | 0.000 | 0.010 | 40.9 |
| 23 | 12 | LOXL2_HUMAN | Lysyl oxidase homolog 2 | 0.002 | 0.101 | 40.5 |
| 2 | 1 | HMCN1_HUMAN | Hemicentin-1 | 0.000 | 0.002 | 40.4 |
| 29 | 14 | POSTN_HUMAN | Periostin | 0.006 | 0.237 | 40.1 |
| 6 | 3 | NPC2_HUMAN | Epididymal secretory protein E1 | 0.000 | 0.013 | 40.1 |
| 20 | 15 | SPRC_HUMAN | SPARC | 0.027 | 1.097 | 40.0 |
| 3 | 1 | UBC9_HUMAN | SUMO-conjugating enzyme UBC9 | 0.000 | 0.003 | 40.0 |
| 39 | 15 | CFAH_HUMAN | Complement factor H | 0.003 | 0.116 | 39.8 |
| 4 | 3 | FABP5_HUMAN | Fatty acid-binding protein, epidermal | 0.000 | 0.018 | 39.7 |
| 35 | 13 | C1S_HUMAN | Complement C1s subcomponent | 0.008 | 0.317 | 39.4 |
| 53 | 14 | CO3A1_HUMAN | Collagen alpha-1(III) chain | 0.015 | 0.589 | 39.4 |
| 2 | 1 | CD81_HUMAN | CD81 antigen | 0.000 | 0.003 | 39.2 |
| 29 | 12 | MA2A1_HUMAN | Alpha-mannosidase 2 | 0.002 | 0.075 | 39.1 |
| 2 | 1 | SGT1_HUMAN | Protein SGT1 homolog {ECO:0000250\|UniProtKB:Q08446} | 0.000 | 0.002 | 39.0 |
| 21 | 15 | LG3BP_HUMAN | Galectin-3-binding protein | 0.007 | 0.268 | 38.5 |
| 10 | 8 | DSG2_HUMAN | Desmoglein-2 | 0.001 | 0.044 | 38.0 |
| 12 | 8 | CO4A1_HUMAN | Collagen alpha-1(IV) chain | 0.002 | 0.064 | 38.0 |
| 4 | 2 | NPTX1_HUMAN | Neuronal pentraxin-1 | 0.000 | 0.007 | 38.0 |
| 4 | 3 | SRPX_HUMAN | Sushi repeat-containing protein SRPX | 0.000 | 0.016 | 37.9 |
| 23 | 12 | GRP78_HUMAN | 78 kDa glucose-regulated protein | 0.005 | 0.174 | 37.7 |
| 1 | 1 | SUMO4_HUMAN | Small ubiquitin-related modifier 4 | 0.000 | 0.002 | 37.7 |
| 15 | 10 | LUM_HUMAN | Lumican | 0.009 | 0.351 | 37.5 |
| 4 | 2 | HNRPD_HUMAN | Heterogeneous nuclear ribonucleoprotein D0 | 0.000 | 0.014 | 37.5 |
| 3 | 3 | PA2G4_HUMAN | Proliferation-associated protein 2G4 | 0.000 | 0.011 | 37.3 |
| 18 | 15 | PLOD2_HUMAN | Procollagen-lysine,2-oxoglutarate 5-dioxygenase 2 | 0.003 | 0.102 | 37.2 |
| 19 | 15 | GALT2_HUMAN | Polypeptide N-acetylgalactosaminyltransferase 2 | 0.003 | 0.102 | 37.2 |
| 14 | 11 | HTRA1_HUMAN | Serine protease HTRA1 | 0.002 | 0.076 | 37.1 |
| 14 | 9 | UCHL1_HUMAN | Ubiquitin carboxyl-terminal hydrolase isozyme L1 | 0.004 | 0.156 | 37.1 |
| 5 | 4 | COPA_HUMAN | Coatomer subunit alpha | 0.000 | 0.014 | 36.9 |
| 36 | 15 | CO6A2_HUMAN | Collagen alpha-2(VI) chain | 0.013 | 0.487 | 36.7 |
| 3 | 3 | NDKA_HUMAN | Nucleoside diphosphate kinase A | 0.001 | 0.021 | 36.5 |
| 3 | 1 | SRP09_HUMAN | Signal recognition particle 9 kDa protein | 0.000 | 0.002 | 36.4 |
| 34 | 15 | NID1_HUMAN | Nidogen-1 | 0.003 | 0.121 | 36.4 |
| 3 | 2 | OAF_HUMAN | Out at first protein homolog | 0.000 | 0.010 | 36.4 |
| 25 | 12 | PDIA1_HUMAN | Protein disulfide-isomerase | 0.004 | 0.153 | 36.1 |
| 14 | 3 | DLDH_HUMAN | Dihydrolipoyl dehydrogenase, mitochondrial | 0.000 | 0.014 | 36.0 |
| 20 | 13 | CALR_HUMAN | Calreticulin | 0.004 | 0.162 | 36.0 |
| 22 | 13 | PCOC1_HUMAN | Procollagen C-endopeptidase enhancer 1 | 0.006 | 0.206 | 35.8 |
| 10 | 7 | EF1A3_HUMAN | Putative elongation factor 1-alpha-like 3 | 0.002 | 0.088 | 35.7 |
| 42 | 15 | LAMC1_HUMAN | Laminin subunit gamma-1 | 0.004 | 0.151 | 35.7 |
| 10 | 7 | CD248_HUMAN | Endosialin | 0.002 | 0.059 | 35.7 |
| 6 | 3 | NUDC_HUMAN | Nuclear migration protein nudC | 0.000 | 0.009 | 35.7 |
| 16 | 11 | SERPH_HUMAN | Serpin H1 | 0.002 | 0.079 | 35.5 |
| 22 | 12 | FSCN1_HUMAN | Fascin | 0.003 | 0.114 | 35.3 |
| 8 | 6 | BMP1_HUMAN | Bone morphogenetic protein 1 | 0.001 | 0.037 | 35.3 |
| 6 | 4 | PSA3_HUMAN | Proteasome subunit alpha type-3 | 0.001 | 0.028 | 34.8 |
| 15 | 10 | MA1A1_HUMAN | Mannosyl-oligosaccharide 1,2-alpha-mannosidase IA | 0.002 | 0.085 | 34.8 |
| 8 | 7 | GGH_HUMAN | Gamma-glutamyl hydrolase | 0.002 | 0.055 | 34.7 |
| 18 | 14 | IBP7_HUMAN | Insulin-like growth factor-binding protein 7 | 0.026 | 0.890 | 34.7 |
| 11 | 6 | PTK7_HUMAN | Inactive tyrosine-protein kinase 7 | 0.001 | 0.036 | 34.7 |
| 19 | 14 | CBPA4_HUMAN | Carboxypeptidase A4 | 0.006 | 0.217 | 34.6 |
| 3 | 1 | RAB1A_HUMAN | Ras-related protein Rab-1A | 0.000 | 0.006 | 34.6 |
| 3 | 2 | COGA1_HUMAN | Collagen alpha-1(XVI) chain | 0.000 | 0.007 | 34.6 |
| 6 | 4 | AP2B1_HUMAN | AP-2 complex subunit beta | 0.000 | 0.010 | 34.5 |
| 2 | 2 | MFAP2_HUMAN | Microfibrillar-associated protein 2 | 0.000 | 0.008 | 34.4 |
| 33 | 15 | ENOA_HUMAN | Alpha-enolase | 0.022 | 0.743 | 34.3 |
| 19 | 14 | MDHM_HUMAN | Malate dehydrogenase, mitochondrial | 0.006 | 0.218 | 34.2 |
| 16 | 7 | GDIA_HUMAN | Rab GDP dissociation inhibitor alpha | 0.002 | 0.057 | 34.2 |
| 32 | 14 | HSP7C_HUMAN | Heat shock cognate 71 kDa protein | 0.007 | 0.243 | 34.2 |
| 104 | 14 | COCA1_HUMAN | Collagen alpha-1(XII) chain | 0.009 | 0.319 | 33.9 |
| 2 | 1 | RUXE_HUMAN | Small nuclear ribonucleoprotein E | 0.000 | 0.003 | 33.8 |
| 4 | 2 | FBLN4_HUMAN | EGF-containing fibulin-like extracellular matrix protein 2 | 0.000 | 0.007 | 33.7 |
| 4 | 3 | RAN_HUMAN | GTP-binding nuclear protein Ran | 0.001 | 0.026 | 33.7 |
| 7 | 5 | B4GT1_HUMAN | Beta-1,4-galactosyltransferase 1 | 0.001 | 0.044 | 33.6 |
| 2 | 1 | PLD3_HUMAN | Phospholipase D3 | 0.000 | 0.002 | 33.6 |
| 39 | 12 | LTBP2_HUMAN | Latent-transforming growth factor beta-binding protein 2 | 0.004 | 0.136 | 33.5 |
| 4 | 2 | FPPS_HUMAN | Farnesyl pyrophosphate synthase | 0.000 | 0.008 | 33.4 |
| 29 | 11 | LAMA4_HUMAN | Laminin subunit alpha-4 | 0.002 | 0.067 | 33.3 |
| 4 | 2 | PPIC_HUMAN | Peptidyl-prolyl cis-trans isomerase C | 0.001 | 0.017 | 33.3 |
| 3 | 1 | MIF_HUMAN | Macrophage migration inhibitory factor | 0.001 | 0.033 | 33.3 |
| 23 | 11 | EMIL1_HUMAN | EMILIN-1 | 0.002 | 0.065 | 33.0 |
| 3 | 1 | FCL_HUMAN | GDP-L-fucose synthase | 0.000 | 0.003 | 32.8 |
| 5 | 2 | SERB_HUMAN | Phosphoserine phosphatase | 0.000 | 0.008 | 32.6 |
| 18 | 11 | 1433E_HUMAN | 14-3-3 protein epsilon | 0.005 | 0.165 | 32.4 |
| 23 | 14 | CH3L1_HUMAN | Chitinase-3-like protein 1 | 0.029 | 0.938 | 32.4 |
| 16 | 15 | TPA_HUMAN | Tissue-type plasminogen activator | 0.003 | 0.106 | 32.4 |
| 4 | 4 | MYDGF_HUMAN | Myeloid-derived growth factor {ECO:0000303\|PubMed:25581518, ECO:0000312\|HGNC:HGNC:16948} | 0.002 | 0.058 | 32.1 |
| 3 | 2 | RBBP4_HUMAN | Histone-binding protein RBBP4 | 0.000 | 0.011 | 32.1 |
| 18 | 13 | LKHA4_HUMAN | Leukotriene A-4 hydrolase | 0.002 | 0.049 | 32.1 |
| 1 | 1 | RS10_HUMAN | 40S ribosomal protein S10 | 0.000 | 0.001 | 32.1 |
| 4 | 3 | TSP3_HUMAN | Thrombospondin-3 | 0.000 | 0.007 | 31.8 |
| 3 | 2 | SGCE_HUMAN | Epsilon-sarcoglycan | 0.000 | 0.004 | 31.8 |
| 14 | 13 | IDHC_HUMAN | Isocitrate dehydrogenase [NADP] cytoplasmic | 0.002 | 0.077 | 31.7 |
| 6 | 3 | RL10A_HUMAN | 60S ribosomal protein L10a | 0.001 | 0.031 | 31.7 |
| 41 | 15 | KPYM_HUMAN | Pyruvate kinase PKM | 0.019 | 0.594 | 31.6 |
| 5 | 5 | HEXA_HUMAN | Beta-hexosaminidase subunit alpha | 0.001 | 0.022 | 31.6 |
| 3 | 3 | 4F2_HUMAN | 4F2 cell-surface antigen heavy chain | 0.000 | 0.011 | 31.6 |
| 7 | 5 | CATZ_HUMAN | Cathepsin Z | 0.001 | 0.037 | 31.6 |
| 26 | 13 | TKT_HUMAN | Transketolase | 0.009 | 0.291 | 31.4 |
| 25 | 12 | PGK1_HUMAN | Phosphoglycerate kinase 1 | 0.006 | 0.179 | 31.4 |
| 27 | 12 | GDIB_HUMAN | Rab GDP dissociation inhibitor beta | 0.004 | 0.123 | 31.2 |
| 1 | 1 | CALX_HUMAN | Calnexin | 0.000 | 0.002 | 31.2 |
| 4 | 1 | HGF_HUMAN | Hepatocyte growth factor | 0.000 | 0.003 | 31.0 |
| 2 | 2 | SC22B_HUMAN | Vesicle-trafficking protein SEC22b | 0.000 | 0.003 | 30.9 |
| 6 | 4 | GLGB_HUMAN | 1,4-alpha-glucan-branching enzyme | 0.001 | 0.017 | 30.9 |
| 1 | 1 | LTOR3_HUMAN | Ragulator complex protein LAMTOR3 | 0.000 | 0.002 | 30.8 |
| 12 | 8 | HSP74_HUMAN | Heat shock 70 kDa protein 4 | 0.001 | 0.038 | 30.8 |
| 11 | 8 | ROA1_HUMAN | Heterogeneous nuclear ribonucleoprotein A1 | 0.004 | 0.110 | 30.5 |
| 37 | 13 | PAPP1_HUMAN | Pappalysin-1 | 0.003 | 0.101 | 30.4 |
| 5 | 5 | RSU1_HUMAN | Ras suppressor protein 1 | 0.001 | 0.017 | 30.4 |
| 21 | 12 | CD109_HUMAN | CD109 antigen | 0.002 | 0.049 | 30.4 |
| 25 | 15 | G6PI_HUMAN | Glucose-6-phosphate isomerase | 0.007 | 0.199 | 30.3 |
| 17 | 13 | CSPG2_HUMAN | Versican core protein | 0.004 | 0.111 | 30.3 |
| 22 | 12 | EXT2_HUMAN | Exostosin-2 | 0.003 | 0.077 | 30.2 |
| 14 | 8 | GSHB_HUMAN | Glutathione synthetase | 0.001 | 0.035 | 30.2 |
| 88 | 15 | CO1A1_HUMAN | Collagen alpha-1(I) chain | 0.071 | 2.125 | 30.1 |
| 12 | 7 | DPYL3_HUMAN | Dihydropyrimidinase-related protein 3 | 0.001 | 0.037 | 29.9 |
| 13 | 9 | HEXB_HUMAN | Beta-hexosaminidase subunit beta | 0.002 | 0.047 | 29.9 |
| 8 | 7 | PDIA6_HUMAN | Protein disulfide-isomerase A6 | 0.001 | 0.033 | 29.8 |
| 27 | 15 | VINC_HUMAN | Vinculin | 0.004 | 0.116 | 29.7 |
| 4 | 2 | FRIL_HUMAN | Ferritin light chain | 0.000 | 0.010 | 29.7 |
| 3 | 2 | ELAV1_HUMAN | ELAV-like protein 1 | 0.000 | 0.005 | 29.6 |
| 6 | 4 | CAPZB_HUMAN | F-actin-capping protein subunit beta | 0.000 | 0.014 | 29.5 |
| 14 | 10 | PEDF_HUMAN | Pigment epithelium-derived factor | 0.003 | 0.085 | 29.4 |
| 4 | 2 | PSME1_HUMAN | Proteasome activator complex subunit 1 | 0.000 | 0.007 | 29.4 |
| 2 | 2 | GLRX3_HUMAN | Glutaredoxin-3 | 0.000 | 0.008 | 29.4 |
| 27 | 15 | ALDOA_HUMAN | Fructose-bisphosphate aldolase A | 0.012 | 0.345 | 29.4 |
| 6 | 2 | LGUL_HUMAN | Lactoylglutathione lyase | 0.000 | 0.013 | 29.3 |
| 6 | 5 | GANAB_HUMAN | Neutral alpha-glucosidase AB | 0.001 | 0.018 | 29.3 |
| 53 | 12 | FLNB_HUMAN | Filamin-B | 0.002 | 0.070 | 29.1 |
| 7 | 4 | TFR1_HUMAN | Transferrin receptor protein 1 | 0.000 | 0.009 | 29.1 |
| 19 | 13 | TERA_HUMAN | Transitional endoplasmic reticulum ATPase | 0.002 | 0.049 | 29.1 |
| 9 | 2 | PTBP1_HUMAN | Polypyrimidine tract-binding protein 1 | 0.000 | 0.010 | 29.1 |
| 22 | 13 | SPON2_HUMAN | Spondin-2 | 0.035 | 1.002 | 29.0 |
| 7 | 3 | IMB1_HUMAN | Importin subunit beta-1 | 0.000 | 0.008 | 28.8 |
| 13 | 11 | GSTP1_HUMAN | Glutathione S-transferase P | 0.007 | 0.197 | 28.7 |
| 18 | 8 | AGRIN_HUMAN | Agrin | 0.001 | 0.033 | 28.6 |
| 19 | 13 | LDHB_HUMAN | L-lactate dehydrogenase B chain | 0.012 | 0.340 | 28.6 |
| 2 | 2 | LIS1_HUMAN | Platelet-activating factor acetylhydrolase IB subunit alpha {ECO:0000255\|HAMAP-Rule:MF_03141} | 0.000 | 0.008 | 28.5 |
| 1 | 1 | PA1B3_HUMAN | Platelet-activating factor acetylhydrolase IB subunit gamma | 0.000 | 0.002 | 28.5 |
| 6 | 4 | ENPL_HUMAN | Endoplasmin | 0.001 | 0.019 | 28.2 |
| 11 | 10 | NUCB1_HUMAN | Nucleobindin-1 | 0.002 | 0.056 | 28.2 |
| 2 | 2 | ADA19_HUMAN | Disintegrin and metalloproteinase domain-containing protein 19 | 0.000 | 0.012 | 28.2 |
| 25 | 15 | TAGL_HUMAN | Transgelin | 0.026 | 0.717 | 28.1 |
| 1 | 1 | FUCO_HUMAN | Tissue alpha-L-fucosidase | 0.000 | 0.004 | 28.0 |
| 27 | 16 | INHBA_HUMAN | Inhibin beta A chain | 0.010 | 0.280 | 28.0 |
| 7 | 3 | HSPB1_HUMAN | Heat shock protein beta-1 | 0.000 | 0.012 | 28.0 |
| 26 | 13 | LDHA_HUMAN | L-lactate dehydrogenase A chain | 0.026 | 0.713 | 27.9 |
| 2 | 2 | ARC1B_HUMAN | Actin-related protein 2/3 complex subunit 1B | 0.000 | 0.006 | 27.8 |
| 12 | 7 | AMD_HUMAN | Peptidyl-glycine alpha-amidating monooxygenase | 0.001 | 0.030 | 27.7 |
| 1 | 1 | ROAA_HUMAN | Heterogeneous nuclear ribonucleoprotein A/B | 0.000 | 0.005 | 27.6 |
| 8 | 5 | DPYL2_HUMAN | Dihydropyrimidinase-related protein 2 | 0.001 | 0.016 | 27.5 |
| 3 | 2 | AN32B_HUMAN | Acidic leucine-rich nuclear phosphoprotein 32 family member B | 0.000 | 0.005 | 27.4 |
| 16 | 12 | AMPB_HUMAN | Aminopeptidase B | 0.002 | 0.065 | 27.4 |
| 6 | 5 | PSB2_HUMAN | Proteasome subunit beta type-2 | 0.001 | 0.039 | 27.3 |
| 13 | 11 | CLC11_HUMAN | C-type lectin domain family 11 member A | 0.003 | 0.081 | 27.2 |
| 16 | 12 | PCD10_HUMAN | Protocadherin-10 | 0.002 | 0.054 | 27.2 |
| 5 | 2 | ECI1_HUMAN | Enoyl-CoA delta isomerase 1, mitochondrial | 0.000 | 0.008 | 27.1 |
| 16 | 13 | MDHC_HUMAN | Malate dehydrogenase, cytoplasmic | 0.007 | 0.189 | 27.0 |
| 9 | 6 | IBP5_HUMAN | Insulin-like growth factor-binding protein 5 | 0.001 | 0.029 | 26.9 |
| 13 | 11 | TAGL2_HUMAN | Transgelin-2 | 0.003 | 0.091 | 26.9 |
| 9 | 4 | ARP3_HUMAN | Actin-related protein 3 | 0.001 | 0.015 | 26.8 |
| 10 | 9 | AK1A1_HUMAN | Alcohol dehydrogenase [NADP(+)] | 0.001 | 0.033 | 26.7 |
| 10 | 6 | FSTL1_HUMAN | Follistatin-related protein 1 | 0.001 | 0.025 | 26.6 |
| 1 | 1 | BAF_HUMAN | Barrier-to-autointegration factor | 0.000 | 0.001 | 26.5 |
| 7 | 3 | UGPA_HUMAN | UTP--glucose-1-phosphate uridylyltransferase | 0.000 | 0.007 | 26.4 |
| 1 | 1 | KAT3_HUMAN | Kynurenine--oxoglutarate transaminase 3 | 0.000 | 0.002 | 26.2 |
| 15 | 11 | PRDX6_HUMAN | Peroxiredoxin-6 | 0.003 | 0.090 | 26.2 |
| 1 | 2 | GLO2_HUMAN | Hydroxyacylglutathione hydrolase, mitochondrial {ECO:0000303\|PubMed:15117945} | 0.000 | 0.003 | 26.2 |
| 16 | 11 | DPP3_HUMAN | Dipeptidyl peptidase 3 | 0.002 | 0.043 | 26.1 |
| 5 | 1 | ARF1_HUMAN | ADP-ribosylation factor 1 | 0.000 | 0.002 | 26.0 |
| 21 | 6 | XYLT1_HUMAN | Xylosyltransferase 1 | 0.001 | 0.028 | 25.9 |
| 2 | 1 | MA1B1_HUMAN | Endoplasmic reticulum mannosyl-oligosaccharide 1,2-alpha-mannosidase | 0.000 | 0.002 | 25.9 |
| 1 | 1 | S10AG_HUMAN | Protein S100-A16 | 0.000 | 0.001 | 25.8 |
| 4 | 2 | GALT6_HUMAN | Polypeptide N-acetylgalactosaminyltransferase 6 | 0.000 | 0.005 | 25.8 |
| 10 | 7 | CATD_HUMAN | Cathepsin D | 0.002 | 0.039 | 25.7 |
| 49 | 15 | VIME_HUMAN | Vimentin | 0.029 | 0.750 | 25.6 |
| 7 | 6 | PDLI5_HUMAN | PDZ and LIM domain protein 5 | 0.001 | 0.019 | 25.5 |
| 14 | 9 | HS71B_HUMAN | Heat shock 70 kDa protein 1B {ECO:0000312\|HGNC:HGNC:5233} | 0.003 | 0.064 | 25.4 |
| 7 | 5 | ERAP1_HUMAN | Endoplasmic reticulum aminopeptidase 1 | 0.000 | 0.011 | 25.4 |
| 5 | 4 | TXD17_HUMAN | Thioredoxin domain-containing protein 17 | 0.001 | 0.033 | 25.4 |
| 2 | 1 | STC1_HUMAN | Stanniocalcin-1 | 0.000 | 0.002 | 25.2 |
| 1 | 1 | CUTA_HUMAN | Protein CutA | 0.000 | 0.004 | 25.2 |
| 15 | 14 | G3P_HUMAN | Glyceraldehyde-3-phosphate dehydrogenase | 0.011 | 0.285 | 25.2 |
| 3 | 2 | SEPR_HUMAN | Prolyl endopeptidase FAP {ECO:0000305} | 0.000 | 0.005 | 25.1 |
| 18 | 14 | TPIS_HUMAN | Triosephosphate isomerase | 0.021 | 0.521 | 25.1 |
| 18 | 10 | TRXR1_HUMAN | Thioredoxin reductase 1, cytoplasmic | 0.002 | 0.057 | 24.9 |
| 7 | 3 | ATL1_HUMAN | ADAMTS-like protein 1 | 0.001 | 0.017 | 24.8 |
| 4 | 2 | VP26A_HUMAN | Vacuolar protein sorting-associated protein 26A | 0.000 | 0.007 | 24.7 |
| 1 | 1 | STRP1_HUMAN | Striatin-interacting protein 1 | 0.000 | 0.001 | 24.6 |
| 1 | 1 | ERP44_HUMAN | Endoplasmic reticulum resident protein 44 | 0.000 | 0.002 | 24.6 |
| 5 | 1 | PURA2_HUMAN | Adenylosuccinate synthetase isozyme 2 {ECO:0000255\|HAMAP-Rule:MF_03127} | 0.000 | 0.002 | 24.6 |
| 13 | 6 | CBR1_HUMAN | Carbonyl reductase [NADPH] 1 | 0.002 | 0.038 | 24.5 |
| 8 | 5 | GLU2B_HUMAN | Glucosidase 2 subunit beta | 0.001 | 0.022 | 24.5 |
| 4 | 4 | PROF2_HUMAN | Profilin-2 | 0.000 | 0.011 | 24.4 |
| 3 | 2 | TPM3_HUMAN | Tropomyosin alpha-3 chain | 0.000 | 0.004 | 24.2 |
| 17 | 11 | SODM_HUMAN | Superoxide dismutase [Mn], mitochondrial | 0.003 | 0.074 | 24.2 |
| 14 | 9 | PRDX1_HUMAN | Peroxiredoxin-1 | 0.007 | 0.161 | 24.0 |
| 22 | 13 | WDR1_HUMAN | WD repeat-containing protein 1 | 0.005 | 0.126 | 23.9 |
| 33 | 14 | EF2_HUMAN | Elongation factor 2 | 0.006 | 0.145 | 23.9 |
| 9 | 6 | B4GA1_HUMAN | Beta-1,4-glucuronyltransferase 1 {ECO:0000303\|PubMed:25279697, ECO:0000303\|PubMed:25279699, ECO:0000312\|HGNC:HGNC:15685} | 0.002 | 0.038 | 23.8 |
| 3 | 1 | U2AF2_HUMAN | Splicing factor U2AF 65 kDa subunit | 0.000 | 0.004 | 23.8 |
| 9 | 3 | ARPC2_HUMAN | Actin-related protein 2/3 complex subunit 2 | 0.001 | 0.018 | 23.6 |
| 11 | 8 | QPCT_HUMAN | Glutaminyl-peptide cyclotransferase | 0.002 | 0.045 | 23.6 |
| 18 | 10 | ROA2_HUMAN | Heterogeneous nuclear ribonucleoproteins A2/B1 | 0.003 | 0.077 | 23.6 |
| 1 | 1 | SEPT9_HUMAN | Septin-9 | 0.000 | 0.002 | 23.6 |
| 6 | 4 | 6PGD_HUMAN | 6-phosphogluconate dehydrogenase, decarboxylating | 0.001 | 0.014 | 23.5 |
| 13 | 9 | TALDO_HUMAN | Transaldolase | 0.002 | 0.057 | 23.4 |
| 4 | 1 | CALU_HUMAN | Calumenin | 0.000 | 0.009 | 23.3 |
| 8 | 4 | CNDP2_HUMAN | Cytosolic non-specific dipeptidase | 0.001 | 0.017 | 23.1 |
| 1 | 1 | TFPI1_HUMAN | Tissue factor pathway inhibitor | 0.000 | 0.002 | 23.1 |
| 12 | 11 | CYTC_HUMAN | Cystatin-C | 0.015 | 0.341 | 23.0 |
| 2 | 2 | LXN_HUMAN | Latexin | 0.000 | 0.007 | 22.7 |
| 6 | 4 | SRPX2_HUMAN | Sushi repeat-containing protein SRPX2 | 0.001 | 0.021 | 22.6 |
| 23 | 12 | LRP1_HUMAN | Prolow-density lipoprotein receptor-related protein 1 | 0.002 | 0.037 | 22.6 |
| 9 | 7 | PSA1_HUMAN | Proteasome subunit alpha type-1 | 0.002 | 0.051 | 22.5 |
| 1 | 1 | HIBCH_HUMAN | 3-hydroxyisobutyryl-CoA hydrolase, mitochondrial | 0.000 | 0.002 | 22.5 |
| 6 | 4 | PRDX3_HUMAN | Thioredoxin-dependent peroxide reductase, mitochondrial | 0.001 | 0.017 | 22.4 |
| 5 | 3 | PVRL2_HUMAN | Nectin-2 | 0.000 | 0.009 | 22.4 |
| 2 | 2 | CYTB_HUMAN | Cystatin-B | 0.000 | 0.010 | 22.4 |
| 16 | 12 | PPIA_HUMAN | Peptidyl-prolyl cis-trans isomerase A | 0.010 | 0.226 | 22.4 |
| 2 | 2 | 1A66_HUMAN | HLA class I histocompatibility antigen, A-66 alpha chain | 0.000 | 0.004 | 22.4 |
| 11 | 7 | IL6_HUMAN | Interleukin-6 | 0.006 | 0.140 | 22.4 |
| 3 | 2 | KAD1_HUMAN | Adenylate kinase isoenzyme 1 {ECO:0000255\|HAMAP-Rule:MF_03171} | 0.000 | 0.006 | 22.4 |
| 42 | 10 | PLEC_HUMAN | Plectin | 0.002 | 0.043 | 22.3 |
| 10 | 10 | CH60_HUMAN | 60 kDa heat shock protein, mitochondrial | 0.001 | 0.032 | 22.3 |
| 3 | 3 | SYK_HUMAN | Lysine--tRNA ligase | 0.000 | 0.007 | 22.3 |
| 3 | 1 | LDLR_HUMAN | Low-density lipoprotein receptor | 0.000 | 0.003 | 22.2 |
| 6 | 3 | CAN2_HUMAN | Calpain-2 catalytic subunit | 0.000 | 0.007 | 22.1 |
| 3 | 2 | TPP1_HUMAN | Tripeptidyl-peptidase 1 | 0.000 | 0.002 | 22.1 |
| 10 | 8 | CLIC1_HUMAN | Chloride intracellular channel protein 1 | 0.003 | 0.076 | 22.1 |
| 2 | 1 | BPNT1_HUMAN | 3'(2'),5'-bisphosphate nucleotidase 1 | 0.000 | 0.003 | 22.0 |
| 2 | 2 | ITB1_HUMAN | Integrin beta-1 | 0.000 | 0.004 | 22.0 |
| 17 | 11 | EXT1_HUMAN | Exostosin-1 | 0.003 | 0.068 | 21.9 |
| 29 | 12 | TLN1_HUMAN | Talin-1 | 0.002 | 0.046 | 21.9 |
| 12 | 10 | CLUS_HUMAN | Clusterin | 0.004 | 0.088 | 21.8 |
| 6 | 5 | PSB3_HUMAN | Proteasome subunit beta type-3 | 0.001 | 0.027 | 21.7 |
| 7 | 7 | FUMH_HUMAN | Fumarate hydratase, mitochondrial | 0.001 | 0.024 | 21.6 |
| 3 | 2 | CAPR1_HUMAN | Caprin-1 | 0.000 | 0.005 | 21.6 |
| 13 | 11 | ALDR_HUMAN | Aldose reductase | 0.005 | 0.101 | 21.5 |
| 6 | 4 | SDCB1_HUMAN | Syntenin-1 | 0.001 | 0.012 | 21.5 |
| 2 | 2 | ILF3_HUMAN | Interleukin enhancer-binding factor 3 | 0.000 | 0.008 | 21.4 |
| 2 | 2 | TPM1_HUMAN | Tropomyosin alpha-1 chain | 0.000 | 0.004 | 21.4 |
| 7 | 3 | TPM4_HUMAN | Tropomyosin alpha-4 chain | 0.000 | 0.010 | 21.3 |
| 24 | 10 | ACTG_HUMAN | Actin, cytoplasmic 2 | 0.011 | 0.235 | 21.2 |
| 14 | 12 | MYH9_HUMAN | Myosin-9 | 0.003 | 0.055 | 21.2 |
| 97 | 14 | PGBM_HUMAN | Basement membrane-specific heparan sulfate proteoglycan core protein | 0.013 | 0.267 | 21.1 |
| 7 | 6 | IF6_HUMAN | Eukaryotic translation initiation factor 6 {ECO:0000255\|HAMAP-Rule:MF_03132} | 0.001 | 0.018 | 21.1 |
| 9 | 8 | IPO5_HUMAN | Importin-5 | 0.001 | 0.023 | 21.1 |
| 38 | 13 | CSPG4_HUMAN | Chondroitin sulfate proteoglycan 4 | 0.004 | 0.084 | 21.1 |
| 9 | 7 | MXRA8_HUMAN | Matrix-remodeling-associated protein 8 | 0.002 | 0.049 | 21.0 |
| 19 | 13 | CADH2_HUMAN | Cadherin-2 | 0.005 | 0.103 | 21.0 |
| 16 | 9 | CAP1_HUMAN | Adenylyl cyclase-associated protein 1 | 0.004 | 0.092 | 21.0 |
| 4 | 2 | TFPI2_HUMAN | Tissue factor pathway inhibitor 2 | 0.000 | 0.008 | 21.0 |
| 4 | 1 | 1A69_HUMAN | HLA class I histocompatibility antigen, A-69 alpha chain | 0.000 | 0.001 | 20.9 |
| 10 | 4 | SPB7_HUMAN | Serpin B7 | 0.001 | 0.019 | 20.8 |
| 5 | 3 | ERP29_HUMAN | Endoplasmic reticulum resident protein 29 | 0.000 | 0.009 | 20.8 |
| 15 | 11 | CLIC4_HUMAN | Chloride intracellular channel protein 4 | 0.003 | 0.064 | 20.7 |
| 4 | 3 | T132A_HUMAN | Transmembrane protein 132A | 0.000 | 0.006 | 20.7 |
| 4 | 1 | COPG1_HUMAN | Coatomer subunit gamma-1 | 0.000 | 0.005 | 20.7 |
| 3 | 1 | BCAT1_HUMAN | Branched-chain-amino-acid aminotransferase, cytosolic | 0.000 | 0.004 | 20.7 |
| 2 | 2 | AP2A1_HUMAN | AP-2 complex subunit alpha-1 | 0.000 | 0.005 | 20.7 |
| 3 | 1 | GALNS_HUMAN | N-acetylgalactosamine-6-sulfatase | 0.000 | 0.002 | 20.6 |
| 1 | 1 | PP2BA_HUMAN | Serine/threonine-protein phosphatase 2B catalytic subunit alpha isoform | 0.000 | 0.002 | 20.6 |
| 16 | 13 | PPIB_HUMAN | Peptidyl-prolyl cis-trans isomerase B | 0.024 | 0.495 | 20.4 |
| 2 | 2 | PDGFD_HUMAN | Platelet-derived growth factor D | 0.000 | 0.004 | 20.4 |
| 9 | 8 | CATL1_HUMAN | Cathepsin L1 | 0.002 | 0.038 | 20.4 |
| 7 | 3 | GRP75_HUMAN | Stress-70 protein, mitochondrial | 0.000 | 0.010 | 20.3 |
| 1 | 1 | HEM2_HUMAN | Delta-aminolevulinic acid dehydratase | 0.000 | 0.003 | 20.2 |
| 5 | 2 | UB2V1_HUMAN | Ubiquitin-conjugating enzyme E2 variant 1 | 0.001 | 0.011 | 20.1 |
| 7 | 2 | LAMA2_HUMAN | Laminin subunit alpha-2 | 0.000 | 0.003 | 20.1 |
| 7 | 7 | CSRP1_HUMAN | Cysteine and glycine-rich protein 1 | 0.002 | 0.040 | 20.1 |
| 6 | 3 | GLT10_HUMAN | Polypeptide N-acetylgalactosaminyltransferase 10 | 0.001 | 0.013 | 20.0 |
| 4 | 4 | HNRPC_HUMAN | Heterogeneous nuclear ribonucleoproteins C1/C2 | 0.002 | 0.030 | 19.9 |
| 9 | 6 | EZRI_HUMAN | Ezrin | 0.001 | 0.021 | 19.8 |
| 14 | 10 | NEO1_HUMAN | Neogenin | 0.002 | 0.037 | 19.8 |
| 15 | 14 | SERC_HUMAN | Phosphoserine aminotransferase | 0.005 | 0.099 | 19.8 |
| 2 | 2 | MLP3B_HUMAN | Microtubule-associated proteins 1A/1B light chain 3B | 0.001 | 0.013 | 19.8 |
| 8 | 6 | GLOD4_HUMAN | Glyoxalase domain-containing protein 4 | 0.001 | 0.025 | 19.7 |
| 1 | 1 | PUF60_HUMAN | Poly(U)-binding-splicing factor PUF60 | 0.000 | 0.001 | 19.7 |
| 11 | 8 | CAB45_HUMAN | 45 kDa calcium-binding protein | 0.002 | 0.035 | 19.6 |
| 6 | 3 | THIM_HUMAN | 3-ketoacyl-CoA thiolase, mitochondrial | 0.001 | 0.011 | 19.5 |
| 8 | 6 | PRDX2_HUMAN | Peroxiredoxin-2 | 0.002 | 0.043 | 19.4 |
| 7 | 4 | ATPB_HUMAN | ATP synthase subunit beta, mitochondrial | 0.001 | 0.014 | 19.4 |
| 8 | 5 | DEST_HUMAN | Destrin | 0.003 | 0.063 | 19.4 |
| 5 | 2 | COR1C_HUMAN | Coronin-1C | 0.001 | 0.010 | 19.4 |
| 3 | 1 | FA49B_HUMAN | Protein FAM49B | 0.000 | 0.002 | 19.3 |
| 18 | 13 | TIMP2_HUMAN | Metalloproteinase inhibitor 2 | 0.011 | 0.217 | 19.3 |
| 2 | 1 | RD23B_HUMAN | UV excision repair protein RAD23 homolog B | 0.000 | 0.004 | 19.2 |
| 3 | 3 | FKBP3_HUMAN | Peptidyl-prolyl cis-trans isomerase FKBP3 | 0.000 | 0.008 | 19.2 |
| 6 | 6 | GDIR1_HUMAN | Rho GDP-dissociation inhibitor 1 | 0.004 | 0.073 | 19.1 |
| 28 | 11 | FBN2_HUMAN | Fibrillin-2 | 0.002 | 0.031 | 19.1 |
| 1 | 2 | ZYX_HUMAN | Zyxin | 0.000 | 0.003 | 18.9 |
| 3 | 3 | SCRN1_HUMAN | Secernin-1 | 0.000 | 0.007 | 18.9 |
| 6 | 5 | FLRT2_HUMAN | Leucine-rich repeat transmembrane protein FLRT2 | 0.001 | 0.018 | 18.8 |
| 5 | 4 | GDF15_HUMAN | Growth/differentiation factor 15 | 0.001 | 0.015 | 18.8 |
| 13 | 7 | LEG1_HUMAN | Galectin-1 | 0.006 | 0.122 | 18.7 |
| 17 | 6 | PLOD1_HUMAN | Procollagen-lysine,2-oxoglutarate 5-dioxygenase 1 | 0.001 | 0.022 | 18.7 |
| 5 | 2 | PABP1_HUMAN | Polyadenylate-binding protein 1 | 0.000 | 0.006 | 18.6 |
| 2 | 1 | PA1B2_HUMAN | Platelet-activating factor acetylhydrolase IB subunit beta | 0.000 | 0.003 | 18.6 |
| 14 | 9 | 1433Z_HUMAN | 14-3-3 protein zeta/delta | 0.008 | 0.153 | 18.6 |
| 7 | 4 | PLIN3_HUMAN | Perilipin-3 | 0.001 | 0.017 | 18.5 |
| 1 | 1 | APOA1_HUMAN | Apolipoprotein A-I | 0.000 | 0.003 | 18.5 |
| 16 | 11 | NRP1_HUMAN | Neuropilin-1 | 0.003 | 0.051 | 18.5 |
| 10 | 7 | 6PGL_HUMAN | 6-phosphogluconolactonase | 0.003 | 0.054 | 18.5 |
| 3 | 2 | NEUS_HUMAN | Neuroserpin | 0.000 | 0.004 | 18.4 |
| 3 | 1 | RS28_HUMAN | 40S ribosomal protein S28 | 0.001 | 0.009 | 18.3 |
| 3 | 1 | SAHH_HUMAN | Adenosylhomocysteinase | 0.000 | 0.002 | 18.3 |
| 12 | 7 | GSTO1_HUMAN | Glutathione S-transferase omega-1 | 0.006 | 0.111 | 18.3 |
| 5 | 2 | IMPA1_HUMAN | Inositol monophosphatase 1 | 0.000 | 0.005 | 18.3 |
| 2 | 1 | RCN1_HUMAN | Reticulocalbin-1 | 0.000 | 0.002 | 18.2 |
| 6 | 5 | UBE2N_HUMAN | Ubiquitin-conjugating enzyme E2 N | 0.002 | 0.034 | 18.2 |
| 6 | 3 | ILF2_HUMAN | Interleukin enhancer-binding factor 2 | 0.001 | 0.016 | 18.1 |
| 3 | 2 | METK2_HUMAN | S-adenosylmethionine synthase isoform type-2 | 0.000 | 0.006 | 18.0 |
| 8 | 6 | IF5A1_HUMAN | Eukaryotic translation initiation factor 5A-1 | 0.001 | 0.024 | 18.0 |
| 25 | 11 | FLNC_HUMAN | Filamin-C | 0.002 | 0.038 | 18.0 |
| 6 | 5 | CYC_HUMAN | Cytochrome c | 0.004 | 0.064 | 17.9 |
| 11 | 8 | PSB5_HUMAN | Proteasome subunit beta type-5 | 0.002 | 0.036 | 17.8 |
| 5 | 5 | 1433B_HUMAN | 14-3-3 protein beta/alpha | 0.003 | 0.049 | 17.8 |
| 3 | 1 | SYG_HUMAN | Glycine--tRNA ligase | 0.000 | 0.001 | 17.8 |
| 3 | 3 | LAMB2_HUMAN | Laminin subunit beta-2 | 0.000 | 0.006 | 17.8 |
| 2 | 1 | IFT25_HUMAN | Intraflagellar transport protein 25 homolog | 0.000 | 0.003 | 17.6 |
| 30 | 14 | LMNA_HUMAN | Prelamin-A/C | 0.007 | 0.126 | 17.6 |
| 11 | 8 | CTGF_HUMAN | Connective tissue growth factor | 0.003 | 0.049 | 17.6 |
| 4 | 3 | ANAG_HUMAN | Alpha-N-acetylglucosaminidase | 0.000 | 0.006 | 17.5 |
| 17 | 11 | PSA_HUMAN | Puromycin-sensitive aminopeptidase | 0.002 | 0.041 | 17.5 |
| 5 | 4 | FKBP4_HUMAN | Peptidyl-prolyl cis-trans isomerase FKBP4 | 0.000 | 0.008 | 17.5 |
| 13 | 11 | COTL1_HUMAN | Coactosin-like protein | 0.016 | 0.276 | 17.5 |
| 2 | 2 | RO60_HUMAN | 60 kDa SS-A/Ro ribonucleoprotein | 0.000 | 0.005 | 17.4 |
| 4 | 1 | NTF2_HUMAN | Nuclear transport factor 2 | 0.000 | 0.004 | 17.4 |
| 11 | 8 | PSA4_HUMAN | Proteasome subunit alpha type-4 | 0.002 | 0.043 | 17.3 |
| 3 | 3 | RINI_HUMAN | Ribonuclease inhibitor | 0.000 | 0.007 | 17.3 |
| 11 | 7 | PARK7_HUMAN | Protein deglycase DJ-1 {ECO:0000303\|PubMed:25416785} | 0.004 | 0.077 | 17.3 |
| 12 | 11 | AATC_HUMAN | Aspartate aminotransferase, cytoplasmic | 0.003 | 0.058 | 17.3 |
| 6 | 5 | 1433T_HUMAN | 14-3-3 protein theta | 0.001 | 0.021 | 17.1 |
| 5 | 3 | MARE1_HUMAN | Microtubule-associated protein RP/EB family member 1 | 0.000 | 0.007 | 17.0 |
| 2 | 1 | MMP14_HUMAN | Matrix metalloproteinase-14 | 0.000 | 0.001 | 17.0 |
| 3 | 1 | BLVRB_HUMAN | Flavin reductase (NADPH) | 0.000 | 0.001 | 17.0 |
| 2 | 2 | MT1X_HUMAN | Metallothionein-1X | 0.000 | 0.003 | 17.0 |
| 2 | 1 | CAPG_HUMAN | Macrophage-capping protein | 0.000 | 0.002 | 16.9 |
| 6 | 5 | FAAA_HUMAN | Fumarylacetoacetase | 0.001 | 0.021 | 16.9 |
| 3 | 2 | PCNA_HUMAN | Proliferating cell nuclear antigen | 0.000 | 0.004 | 16.9 |
| 3 | 1 | NNRE_HUMAN | NAD(P)H-hydrate epimerase {ECO:0000255\|HAMAP-Rule:MF_03159} | 0.000 | 0.003 | 16.9 |
| 5 | 2 | COEA1_HUMAN | Collagen alpha-1(XIV) chain | 0.001 | 0.009 | 16.9 |
| 7 | 5 | RL40_HUMAN | Ubiquitin-60S ribosomal protein L40 | 0.014 | 0.242 | 16.7 |
| 3 | 2 | EF1D_HUMAN | Elongation factor 1-delta | 0.001 | 0.012 | 16.6 |
| 5 | 1 | PDLI7_HUMAN | PDZ and LIM domain protein 7 | 0.001 | 0.010 | 16.5 |
| 1 | 1 | ROA3_HUMAN | Heterogeneous nuclear ribonucleoprotein A3 | 0.000 | 0.003 | 16.4 |
| 5 | 4 | DKK1_HUMAN | Dickkopf-related protein 1 | 0.001 | 0.018 | 16.4 |
| 10 | 9 | MATN2_HUMAN | Matrilin-2 | 0.002 | 0.030 | 16.4 |
| 1 | 1 | ITA2_HUMAN | Integrin alpha-2 | 0.000 | 0.001 | 16.3 |
| 9 | 7 | PSA2_HUMAN | Proteasome subunit alpha type-2 | 0.003 | 0.041 | 16.2 |
| 16 | 10 | LAMC2_HUMAN | Laminin subunit gamma-2 | 0.002 | 0.034 | 16.2 |
| 3 | 3 | CBPQ_HUMAN | Carboxypeptidase Q | 0.000 | 0.007 | 16.1 |
| 7 | 4 | TSN_HUMAN | Translin | 0.001 | 0.009 | 16.1 |
| 4 | 2 | MAP1B_HUMAN | Microtubule-associated protein 1B | 0.000 | 0.005 | 16.1 |
| 13 | 6 | CO7A1_HUMAN | Collagen alpha-1(VII) chain | 0.001 | 0.015 | 16.1 |
| 19 | 15 | AATM_HUMAN | Aspartate aminotransferase, mitochondrial | 0.011 | 0.172 | 16.0 |
| 1 | 1 | N2DL2_HUMAN | NKG2D ligand 2 | 0.000 | 0.003 | 15.9 |
| 10 | 7 | CCD80_HUMAN | Coiled-coil domain-containing protein 80 | 0.002 | 0.031 | 15.9 |
| 5 | 3 | ILEU_HUMAN | Leukocyte elastase inhibitor | 0.000 | 0.005 | 15.8 |
| 2 | 2 | MAT2B_HUMAN | Methionine adenosyltransferase 2 subunit beta | 0.000 | 0.003 | 15.8 |
| 1 | 1 | SMD1_HUMAN | Small nuclear ribonucleoprotein Sm D1 | 0.000 | 0.003 | 15.8 |
| 3 | 3 | YBOX1_HUMAN | Nuclease-sensitive element-binding protein 1 | 0.001 | 0.009 | 15.8 |
| 4 | 2 | FAM3C_HUMAN | Protein FAM3C | 0.000 | 0.004 | 15.8 |
| 3 | 2 | CSTN3_HUMAN | Calsyntenin-3 | 0.000 | 0.004 | 15.7 |
| 4 | 3 | SH3L3_HUMAN | SH3 domain-binding glutamic acid-rich-like protein 3 | 0.003 | 0.042 | 15.6 |
| 3 | 3 | HS90A_HUMAN | Heat shock protein HSP 90-alpha | 0.001 | 0.009 | 15.6 |
| 6 | 5 | CXCL6_HUMAN | C-X-C motif chemokine 6 | 0.006 | 0.096 | 15.6 |
| 10 | 5 | HNRPQ_HUMAN | Heterogeneous nuclear ribonucleoprotein Q | 0.001 | 0.016 | 15.6 |
| 9 | 3 | RENR_HUMAN | Renin receptor | 0.000 | 0.006 | 15.6 |
| 2 | 1 | IF2A_HUMAN | Eukaryotic translation initiation factor 2 subunit 1 | 0.000 | 0.003 | 15.5 |
| 6 | 5 | APEX1_HUMAN | DNA-(apurinic or apyrimidinic site) lyase | 0.001 | 0.011 | 15.4 |
| 1 | 1 | MFAP4_HUMAN | Microfibril-associated glycoprotein 4 | 0.000 | 0.002 | 15.4 |
| 6 | 4 | COPE_HUMAN | Coatomer subunit epsilon | 0.001 | 0.009 | 15.4 |
| 6 | 2 | MANF_HUMAN | Mesencephalic astrocyte-derived neurotrophic factor | 0.000 | 0.004 | 15.2 |
| 7 | 3 | CPNS1_HUMAN | Calpain small subunit 1 | 0.001 | 0.015 | 15.1 |
| 1 | 1 | SPEE_HUMAN | Spermidine synthase | 0.000 | 0.003 | 15.1 |
| 3 | 2 | RBM8A_HUMAN | RNA-binding protein 8A | 0.000 | 0.005 | 15.1 |
| 9 | 7 | CANT1_HUMAN | Soluble calcium-activated nucleotidase 1 | 0.001 | 0.021 | 15.1 |
| 7 | 4 | ARPC4_HUMAN | Actin-related protein 2/3 complex subunit 4 | 0.002 | 0.030 | 15.0 |
| 4 | 4 | RL12_HUMAN | 60S ribosomal protein L12 | 0.001 | 0.022 | 15.0 |
| 1 | 1 | TBL1R_HUMAN | F-box-like/WD repeat-containing protein TBL1XR1 | 0.000 | 0.001 | 15.0 |
| 8 | 5 | TGFB1_HUMAN | Transforming growth factor beta-1 | 0.001 | 0.020 | 14.9 |
| 1 | 1 | SEP11_HUMAN | Septin-11 | 0.000 | 0.002 | 14.9 |
| 12 | 4 | NDKB_HUMAN | Nucleoside diphosphate kinase B | 0.003 | 0.042 | 14.9 |
| 3 | 3 | CAN1_HUMAN | Calpain-1 catalytic subunit | 0.001 | 0.008 | 14.8 |
| 2 | 1 | SPB9_HUMAN | Serpin B9 | 0.000 | 0.002 | 14.7 |
| 2 | 1 | PPGB_HUMAN | Lysosomal protective protein | 0.000 | 0.001 | 14.7 |
| 1 | 1 | NUDT5_HUMAN | ADP-sugar pyrophosphatase | 0.000 | 0.003 | 14.7 |
| 2 | 1 | IL11_HUMAN | Interleukin-11 | 0.000 | 0.002 | 14.7 |
| 1 | 1 | EFHD2_HUMAN | EF-hand domain-containing protein D2 | 0.000 | 0.002 | 14.6 |
| 4 | 2 | TCPQ_HUMAN | T-complex protein 1 subunit theta | 0.000 | 0.005 | 14.6 |
| 14 | 12 | SPB6_HUMAN | Serpin B6 | 0.006 | 0.087 | 14.6 |
| 11 | 6 | ACPH_HUMAN | Acylamino-acid-releasing enzyme | 0.001 | 0.020 | 14.5 |
| 11 | 9 | ANXA2_HUMAN | Annexin A2 | 0.002 | 0.025 | 14.4 |
| 15 | 9 | PROF1_HUMAN | Profilin-1 | 0.057 | 0.813 | 14.3 |
| 23 | 12 | GELS_HUMAN | Gelsolin | 0.004 | 0.051 | 14.3 |
| 11 | 8 | PSA6_HUMAN | Proteasome subunit alpha type-6 | 0.003 | 0.039 | 14.3 |
| 6 | 2 | MPRI_HUMAN | Cation-independent mannose-6-phosphate receptor | 0.000 | 0.005 | 14.2 |
| 5 | 4 | KCY_HUMAN | UMP-CMP kinase {ECO:0000255\|HAMAP-Rule:MF_03172} | 0.001 | 0.011 | 14.2 |
| 11 | 10 | PSA7_HUMAN | Proteasome subunit alpha type-7 | 0.005 | 0.076 | 14.2 |
| 10 | 5 | B2MG_HUMAN | Beta-2-microglobulin | 0.017 | 0.236 | 14.2 |
| 1 | 1 | CHSTE_HUMAN | Carbohydrate sulfotransferase 14 | 0.000 | 0.002 | 14.2 |
| 1 | 1 | MXRA5_HUMAN | Matrix-remodeling-associated protein 5 | 0.000 | 0.001 | 14.2 |
| 6 | 2 | COF2_HUMAN | Cofilin-2 | 0.001 | 0.010 | 14.1 |
| 2 | 1 | ITM2B_HUMAN | Integral membrane protein 2B | 0.000 | 0.002 | 14.1 |
| 6 | 3 | PRDX4_HUMAN | Peroxiredoxin-4 | 0.001 | 0.010 | 14.1 |
| 4 | 2 | UBP14_HUMAN | Ubiquitin carboxyl-terminal hydrolase 14 | 0.000 | 0.005 | 14.0 |
| 5 | 1 | VEGFC_HUMAN | Vascular endothelial growth factor C | 0.000 | 0.003 | 14.0 |
| 2 | 1 | ANXA6_HUMAN | Annexin A6 | 0.000 | 0.001 | 13.9 |
| 2 | 1 | ADA12_HUMAN | Disintegrin and metalloproteinase domain-containing protein 12 | 0.000 | 0.003 | 13.9 |
| 38 | 11 | TENA_HUMAN | Tenascin | 0.004 | 0.058 | 13.9 |
| 4 | 3 | ARPC3_HUMAN | Actin-related protein 2/3 complex subunit 3 | 0.001 | 0.011 | 13.9 |
| 4 | 4 | ACTS_HUMAN | Actin, alpha skeletal muscle | 0.003 | 0.048 | 13.8 |
| 10 | 8 | PEBP1_HUMAN | Phosphatidylethanolamine-binding protein 1 | 0.007 | 0.095 | 13.8 |
| 5 | 4 | RLA0_HUMAN | 60S acidic ribosomal protein P0 | 0.001 | 0.017 | 13.7 |
| 5 | 5 | VAT1_HUMAN | Synaptic vesicle membrane protein VAT-1 homolog | 0.001 | 0.014 | 13.7 |
| 2 | 2 | DAG1_HUMAN | Dystroglycan | 0.000 | 0.005 | 13.6 |
| 2 | 1 | CAZA1_HUMAN | F-actin-capping protein subunit alpha-1 | 0.000 | 0.005 | 13.6 |
| 3 | 2 | PICAL_HUMAN | Phosphatidylinositol-binding clathrin assembly protein | 0.000 | 0.003 | 13.6 |
| 2 | 1 | H2AJ_HUMAN | Histone H2A.J | 0.000 | 0.002 | 13.5 |
| 4 | 2 | SC31A_HUMAN | Protein transport protein Sec31A | 0.000 | 0.004 | 13.5 |
| 5 | 4 | NUCL_HUMAN | Nucleolin | 0.001 | 0.015 | 13.5 |
| 1 | 1 | RHG01_HUMAN | Rho GTPase-activating protein 1 | 0.000 | 0.001 | 13.4 |
| 2 | 1 | PCDGK_HUMAN | Protocadherin gamma-C3 | 0.000 | 0.001 | 13.3 |
| 9 | 3 | HPRT_HUMAN | Hypoxanthine-guanine phosphoribosyltransferase | 0.001 | 0.008 | 13.3 |
| 1 | 1 | EPN3_HUMAN | Epsin-3 | 0.000 | 0.001 | 13.2 |
| 14 | 8 | COF1_HUMAN | Cofilin-1 | 0.012 | 0.153 | 13.2 |
| 3 | 2 | CIRBP_HUMAN | Cold-inducible RNA-binding protein | 0.000 | 0.005 | 13.2 |
| 2 | 2 | RADI_HUMAN | Radixin | 0.000 | 0.004 | 13.2 |
| 2 | 2 | VAPA_HUMAN | Vesicle-associated membrane protein-associated protein A | 0.000 | 0.005 | 13.2 |
| 6 | 3 | 1433F_HUMAN | 14-3-3 protein eta | 0.001 | 0.010 | 13.2 |
| 13 | 10 | EF1G_HUMAN | Elongation factor 1-gamma | 0.005 | 0.060 | 13.1 |
| 7 | 5 | CHID1_HUMAN | Chitinase domain-containing protein 1 | 0.001 | 0.015 | 13.1 |
| 9 | 5 | 1433G_HUMAN | 14-3-3 protein gamma | 0.004 | 0.048 | 13.1 |
| 10 | 8 | PSA5_HUMAN | Proteasome subunit alpha type-5 | 0.003 | 0.039 | 13.1 |
| 9 | 7 | TWF2_HUMAN | Twinfilin-2 | 0.001 | 0.019 | 13.1 |
| 9 | 8 | IBP4_HUMAN | Insulin-like growth factor-binding protein 4 | 0.005 | 0.066 | 13.1 |
| 2 | 2 | MFGM_HUMAN | Lactadherin | 0.000 | 0.005 | 13.0 |
| 4 | 1 | ORN_HUMAN | Oligoribonuclease, mitochondrial | 0.000 | 0.006 | 13.0 |
| 2 | 2 | AMPL_HUMAN | Cytosol aminopeptidase | 0.000 | 0.004 | 13.0 |
| 5 | 2 | NAGK_HUMAN | N-acetyl-D-glucosamine kinase | 0.000 | 0.005 | 13.0 |
| 6 | 3 | LMNB2_HUMAN | Lamin-B2 | 0.000 | 0.005 | 12.7 |
| 3 | 3 | SIL1_HUMAN | Nucleotide exchange factor SIL1 | 0.001 | 0.007 | 12.7 |
| 15 | 8 | IQGA1_HUMAN | Ras GTPase-activating-like protein IQGAP1 | 0.002 | 0.023 | 12.6 |
| 1 | 1 | SLIT2_HUMAN | Slit homolog 2 protein | 0.000 | 0.001 | 12.6 |
| 4 | 2 | CNN3_HUMAN | Calponin-3 | 0.000 | 0.003 | 12.6 |
| 2 | 1 | RL5_HUMAN | 60S ribosomal protein L5 | 0.000 | 0.001 | 12.6 |
| 4 | 2 | HEBP2_HUMAN | Heme-binding protein 2 | 0.000 | 0.005 | 12.5 |
| 4 | 1 | PTPRS_HUMAN | Receptor-type tyrosine-protein phosphatase S | 0.000 | 0.002 | 12.4 |
| 15 | 12 | LAMA5_HUMAN | Laminin subunit alpha-5 | 0.002 | 0.023 | 12.3 |
| 12 | 2 | HS90B_HUMAN | Heat shock protein HSP 90-beta | 0.001 | 0.012 | 12.3 |
| 2 | 2 | PARVA_HUMAN | Alpha-parvin | 0.000 | 0.003 | 12.3 |
| 1 | 1 | XYLT2_HUMAN | Xylosyltransferase 2 | 0.000 | 0.002 | 12.3 |
| 2 | 1 | TMOD3_HUMAN | Tropomodulin-3 | 0.000 | 0.001 | 12.3 |
| 10 | 2 | PYGB_HUMAN | Glycogen phosphorylase, brain form | 0.000 | 0.003 | 12.0 |
| 1 | 2 | GRPE1_HUMAN | GrpE protein homolog 1, mitochondrial | 0.000 | 0.003 | 12.0 |
| 3 | 2 | FKB1A_HUMAN | Peptidyl-prolyl cis-trans isomerase FKBP1A | 0.001 | 0.009 | 11.9 |
| 5 | 2 | SH3L1_HUMAN | SH3 domain-binding glutamic acid-rich-like protein | 0.001 | 0.009 | 11.8 |
| 6 | 4 | QORX_HUMAN | Quinone oxidoreductase PIG3 | 0.002 | 0.022 | 11.8 |
| 4 | 2 | COIA1_HUMAN | Collagen alpha-1(XVIII) chain | 0.001 | 0.009 | 11.8 |
| 1 | 1 | UFD1_HUMAN | Ubiquitin fusion degradation protein 1 homolog | 0.000 | 0.003 | 11.7 |
| 1 | 1 | RL30_HUMAN | 60S ribosomal protein L30 | 0.000 | 0.001 | 11.7 |
| 17 | 14 | PGAM1_HUMAN | Phosphoglycerate mutase 1 | 0.014 | 0.160 | 11.6 |
| 7 | 3 | QOR_HUMAN | Quinone oxidoreductase | 0.001 | 0.010 | 11.6 |
| 4 | 2 | G6PD_HUMAN | Glucose-6-phosphate 1-dehydrogenase | 0.000 | 0.005 | 11.6 |
| 1 | 1 | SND1_HUMAN | Staphylococcal nuclease domain-containing protein 1 | 0.000 | 0.003 | 11.5 |
| 2 | 2 | CNN2_HUMAN | Calponin-2 | 0.000 | 0.004 | 11.5 |
| 3 | 2 | VTNC_HUMAN | Vitronectin | 0.002 | 0.020 | 11.5 |
| 3 | 2 | UBP5_HUMAN | Ubiquitin carboxyl-terminal hydrolase 5 | 0.000 | 0.003 | 11.5 |
| 8 | 3 | PSB7_HUMAN | Proteasome subunit beta type-7 | 0.002 | 0.020 | 11.4 |
| 6 | 3 | CH10_HUMAN | 10 kDa heat shock protein, mitochondrial | 0.001 | 0.011 | 11.3 |
| 4 | 2 | GNPI1_HUMAN | Glucosamine-6-phosphate isomerase 1 | 0.001 | 0.007 | 11.3 |
| 6 | 2 | PTPRF_HUMAN | Receptor-type tyrosine-protein phosphatase F | 0.000 | 0.004 | 11.2 |
| 5 | 3 | PSB6_HUMAN | Proteasome subunit beta type-6 | 0.002 | 0.020 | 11.2 |
| 3 | 1 | ARL3_HUMAN | ADP-ribosylation factor-like protein 3 | 0.000 | 0.001 | 11.2 |
| 2 | 2 | CRIM1_HUMAN | Cysteine-rich motor neuron 1 protein | 0.000 | 0.002 | 11.2 |
| 2 | 1 | PHS_HUMAN | Pterin-4-alpha-carbinolamine dehydratase | 0.000 | 0.003 | 10.9 |
| 3 | 2 | SODC_HUMAN | Superoxide dismutase [Cu-Zn] | 0.000 | 0.005 | 10.8 |
| 5 | 3 | VGFR1_HUMAN | Vascular endothelial growth factor receptor 1 | 0.001 | 0.008 | 10.8 |
| 1 | 1 | C1QT1_HUMAN | Complement C1q tumor necrosis factor-related protein 1 | 0.000 | 0.002 | 10.7 |
| 3 | 2 | LRC59_HUMAN | Leucine-rich repeat-containing protein 59 | 0.000 | 0.003 | 10.7 |
| 6 | 3 | UBE2K_HUMAN | Ubiquitin-conjugating enzyme E2 K | 0.001 | 0.009 | 10.7 |
| 3 | 1 | GSHR_HUMAN | Glutathione reductase, mitochondrial | 0.000 | 0.002 | 10.7 |
| 4 | 2 | FST_HUMAN | Follistatin | 0.000 | 0.004 | 10.6 |
| 3 | 2 | ESYT1_HUMAN | Extended synaptotagmin-1 | 0.000 | 0.003 | 10.5 |
| 7 | 2 | ML12A_HUMAN | Myosin regulatory light chain 12A | 0.000 | 0.005 | 10.5 |
| 6 | 7 | DDB1_HUMAN | DNA damage-binding protein 1 | 0.002 | 0.021 | 10.4 |
| 3 | 1 | UFC1_HUMAN | Ubiquitin-fold modifier-conjugating enzyme 1 | 0.000 | 0.003 | 10.3 |
| 3 | 2 | PCBP1_HUMAN | Poly(rC)-binding protein 1 | 0.001 | 0.006 | 10.3 |
| 1 | 1 | MATN3_HUMAN | Matrilin-3 | 0.000 | 0.002 | 10.3 |
| 13 | 6 | COPD_HUMAN | Coatomer subunit delta | 0.002 | 0.021 | 10.3 |
| 10 | 6 | PSB1_HUMAN | Proteasome subunit beta type-1 | 0.003 | 0.034 | 10.3 |
| 2 | 1 | IBP6_HUMAN | Insulin-like growth factor-binding protein 6 | 0.000 | 0.003 | 10.2 |
| 2 | 1 | MTAP_HUMAN | S-methyl-5'-thioadenosine phosphorylase {ECO:0000255\|HAMAP-Rule:MF_03155} | 0.000 | 0.003 | 10.2 |
| 2 | 2 | SYQ_HUMAN | Glutamine--tRNA ligase | 0.000 | 0.003 | 10.2 |
| 6 | 5 | TBB5_HUMAN | Tubulin beta chain | 0.002 | 0.017 | 10.1 |
| 8 | 6 | PLTP_HUMAN | Phospholipid transfer protein | 0.002 | 0.022 | 10.1 |
| 5 | 4 | CFAB_HUMAN | Complement factor B | 0.002 | 0.018 | 10.0 |
| 3 | 3 | GNPTG_HUMAN | N-acetylglucosamine-1-phosphotransferase subunit gamma | 0.001 | 0.008 | 10.0 |
| 1 | 1 | RS12_HUMAN | 40S ribosomal protein S12 | 0.001 | 0.006 | 10.0 |
| 3 | 2 | NEUL_HUMAN | Neurolysin, mitochondrial | 0.000 | 0.003 | 10.0 |
| 2 | 2 | PTGR1_HUMAN | Prostaglandin reductase 1 | 0.000 | 0.004 | 9.9 |
| 2 | 1 | ANM1_HUMAN | Protein arginine N-methyltransferase 1 | 0.000 | 0.002 | 9.7 |
| 3 | 1 | TFG_HUMAN | Protein TFG | 0.000 | 0.004 | 9.7 |
| 1 | 1 | TGM2_HUMAN | Protein-glutamine gamma-glutamyltransferase 2 | 0.000 | 0.002 | 9.7 |
| 6 | 5 | MYL6_HUMAN | Myosin light polypeptide 6 | 0.001 | 0.012 | 9.6 |
| 4 | 2 | RET1_HUMAN | Retinol-binding protein 1 | 0.000 | 0.003 | 9.4 |
| 6 | 6 | K2C8_HUMAN | Keratin, type II cytoskeletal 8 | 0.002 | 0.019 | 9.3 |
| 7 | 6 | PGM2_HUMAN | Phosphoglucomutase-2 | 0.002 | 0.020 | 9.3 |
| 7 | 5 | K1C18_HUMAN | Keratin, type I cytoskeletal 18 | 0.002 | 0.017 | 9.3 |
| 5 | 3 | ANXA1_HUMAN | Annexin A1 | 0.001 | 0.005 | 9.2 |
| 3 | 1 | PAMR1_HUMAN | Inactive serine protease PAMR1 | 0.000 | 0.001 | 9.2 |
| 3 | 3 | SYTC_HUMAN | Threonine--tRNA ligase, cytoplasmic | 0.001 | 0.007 | 9.1 |
| 4 | 4 | P3H1_HUMAN | Prolyl 3-hydroxylase 1 {ECO:0000312\|HGNC:HGNC:19316} | 0.001 | 0.012 | 9.1 |
| 2 | 1 | DTD1_HUMAN | D-tyrosyl-tRNA(Tyr) deacylase 1 | 0.000 | 0.003 | 9.0 |
| 2 | 1 | S10AD_HUMAN | Protein S100-A13 | 0.000 | 0.001 | 8.9 |
| 2 | 2 | LA_HUMAN | Lupus La protein | 0.000 | 0.003 | 8.9 |
| 3 | 1 | DDAH2_HUMAN | N(G),N(G)-dimethylarginine dimethylaminohydrolase 2 | 0.000 | 0.003 | 8.9 |
| 5 | 4 | S10AB_HUMAN | Protein S100-A11 | 0.002 | 0.017 | 8.8 |
| 1 | 1 | SH24A_HUMAN | SH2 domain-containing protein 4A | 0.001 | 0.005 | 8.8 |
| 2 | 2 | LEG3_HUMAN | Galectin-3 | 0.000 | 0.004 | 8.8 |
| 1 | 1 | MMP3_HUMAN | Stromelysin-1 | 0.000 | 0.003 | 8.8 |
| 2 | 1 | ENOG_HUMAN | Gamma-enolase | 0.000 | 0.002 | 8.7 |
| 2 | 1 | LMNB1_HUMAN | Lamin-B1 | 0.000 | 0.001 | 8.7 |
| 1 | 1 | PSB8_HUMAN | Proteasome subunit beta type-8 | 0.000 | 0.003 | 8.6 |
| 2 | 2 | MYL9_HUMAN | Myosin regulatory light polypeptide 9 | 0.000 | 0.003 | 8.5 |
| 8 | 6 | DSC3_HUMAN | Desmocollin-3 | 0.006 | 0.054 | 8.5 |
| 5 | 3 | FAHD1_HUMAN | Acylpyruvase FAHD1, mitochondrial | 0.001 | 0.007 | 8.5 |
| 3 | 1 | ARSA_HUMAN | Arylsulfatase A | 0.000 | 0.001 | 8.5 |
| 3 | 1 | COPB2_HUMAN | Coatomer subunit beta' | 0.000 | 0.002 | 8.4 |
| 6 | 5 | RB11B_HUMAN | Ras-related protein Rab-11B | 0.003 | 0.021 | 8.4 |
| 3 | 3 | IDI1_HUMAN | Isopentenyl-diphosphate Delta-isomerase 1 | 0.000 | 0.004 | 8.3 |
| 4 | 3 | TSG6_HUMAN | Tumor necrosis factor-inducible gene 6 protein | 0.002 | 0.017 | 8.2 |
| 5 | 2 | THIO_HUMAN | Thioredoxin | 0.001 | 0.012 | 8.2 |
| 5 | 1 | MGT5A_HUMAN | Alpha-1,6-mannosylglycoprotein 6-beta-N-acetylglucosaminyltransferase A | 0.000 | 0.002 | 8.1 |
| 1 | 1 | SMD3_HUMAN | Small nuclear ribonucleoprotein Sm D3 | 0.001 | 0.004 | 8.1 |
| 3 | 2 | SAP3_HUMAN | Ganglioside GM2 activator | 0.001 | 0.005 | 8.1 |
| 1 | 1 | FIS1_HUMAN | Mitochondrial fission 1 protein | 0.000 | 0.000 | 8.0 |
| 2 | 2 | FHL1_HUMAN | Four and a half LIM domains protein 1 | 0.001 | 0.006 | 8.0 |
| 1 | 1 | TARA_HUMAN | TRIO and F-actin-binding protein | 0.000 | 0.001 | 8.0 |
| 2 | 1 | HBD_HUMAN | Hemoglobin subunit delta | 0.004 | 0.029 | 8.0 |
| 1 | 1 | ARF4_HUMAN | ADP-ribosylation factor 4 | 0.000 | 0.001 | 7.9 |
| 2 | 2 | GNPI2_HUMAN | Glucosamine-6-phosphate isomerase 2 | 0.000 | 0.002 | 7.8 |
| 3 | 3 | EF1B_HUMAN | Elongation factor 1-beta | 0.001 | 0.008 | 7.8 |
| 2 | 2 | MTND_HUMAN | 1,2-dihydroxy-3-keto-5-methylthiopentene dioxygenase {ECO:0000255\|HAMAP-Rule:MF_03154} | 0.000 | 0.004 | 7.7 |
| 1 | 1 | ISLR_HUMAN | Immunoglobulin superfamily containing leucine-rich repeat protein | 0.000 | 0.001 | 7.7 |
| 18 | 15 | CAD11_HUMAN | Cadherin-11 | 0.021 | 0.162 | 7.7 |
| 2 | 2 | NIBL1_HUMAN | Niban-like protein 1 | 0.001 | 0.004 | 7.7 |
| 3 | 1 | DCTN3_HUMAN | Dynactin subunit 3 | 0.000 | 0.001 | 7.6 |
| 6 | 5 | FSTL5_HUMAN | Follistatin-related protein 5 | 0.001 | 0.011 | 7.5 |
| 7 | 4 | GROA_HUMAN | Growth-regulated alpha protein | 0.005 | 0.035 | 7.5 |
| 5 | 2 | ACOC_HUMAN | Cytoplasmic aconitate hydratase | 0.001 | 0.004 | 7.4 |
| 4 | 1 | NPS3A_HUMAN | Protein NipSnap homolog 3A | 0.000 | 0.002 | 7.3 |
| 1 | 1 | CDC42_HUMAN | Cell division control protein 42 homolog | 0.000 | 0.001 | 7.2 |
| 4 | 3 | ITIH2_HUMAN | Inter-alpha-trypsin inhibitor heavy chain H2 | 0.002 | 0.012 | 7.1 |
| 3 | 2 | LASP1_HUMAN | LIM and SH3 domain protein 1 | 0.001 | 0.004 | 7.1 |
| 2 | 2 | HNRPR_HUMAN | Heterogeneous nuclear ribonucleoprotein R | 0.000 | 0.003 | 7.1 |
| 13 | 5 | COBA1_HUMAN | Collagen alpha-1(XI) chain | 0.002 | 0.013 | 7.0 |
| 4 | 4 | MGN2_HUMAN | Protein mago nashi homolog 2 | 0.001 | 0.006 | 6.8 |
| 8 | 5 | AMPN_HUMAN | Aminopeptidase N | 0.002 | 0.013 | 6.7 |
| 7 | 2 | HYI_HUMAN | Putative hydroxypyruvate isomerase | 0.001 | 0.003 | 6.6 |
| 1 | 1 | UFM1_HUMAN | Ubiquitin-fold modifier 1 | 0.000 | 0.003 | 6.6 |
| 3 | 2 | CNN1_HUMAN | Calponin-1 | 0.000 | 0.003 | 6.4 |
| 2 | 1 | CDC37_HUMAN | Hsp90 co-chaperone Cdc37 | 0.000 | 0.002 | 6.4 |
| 2 | 2 | MTPN_HUMAN | Myotrophin | 0.000 | 0.002 | 6.4 |
| 1 | 1 | FAS_HUMAN | Fatty acid synthase | 0.000 | 0.001 | 6.4 |
| 2 | 2 | THOP1_HUMAN | Thimet oligopeptidase | 0.001 | 0.004 | 6.3 |
| 10 | 5 | A4_HUMAN | Amyloid beta A4 protein | 0.003 | 0.017 | 6.3 |
| 1 | 1 | NQO2_HUMAN | Ribosyldihydronicotinamide dehydrogenase [quinone] | 0.000 | 0.001 | 6.3 |
| 11 | 7 | TBA1B_HUMAN | Tubulin alpha-1B chain | 0.004 | 0.025 | 6.2 |
| 2 | 1 | 2AAA_HUMAN | Serine/threonine-protein phosphatase 2A 65 kDa regulatory subunit A alpha isoform | 0.000 | 0.001 | 6.1 |
| 13 | 6 | SPTN1_HUMAN | Spectrin alpha chain, non-erythrocytic 1 | 0.001 | 0.006 | 6.0 |
| 2 | 1 | CPSF5_HUMAN | Cleavage and polyadenylation specificity factor subunit 5 | 0.000 | 0.001 | 5.6 |
| 2 | 1 | PHP14_HUMAN | 14 kDa phosphohistidine phosphatase | 0.000 | 0.001 | 5.6 |
| 1 | 1 | INPP_HUMAN | Inositol polyphosphate 1-phosphatase | 0.000 | 0.001 | 5.6 |
| 4 | 4 | GREM1_HUMAN | Gremlin-1 | 0.002 | 0.011 | 5.6 |
| 3 | 1 | HNRPK_HUMAN | Heterogeneous nuclear ribonucleoprotein K | 0.000 | 0.001 | 5.5 |
| 1 | 1 | RAC1_HUMAN | Ras-related C3 botulinum toxin substrate 1 | 0.000 | 0.001 | 5.4 |
| 1 | 1 | HXK1_HUMAN | Hexokinase-1 | 0.000 | 0.001 | 5.3 |
| 1 | 1 | HEM6_HUMAN | Oxygen-dependent coproporphyrinogen-III oxidase, mitochondrial | 0.000 | 0.001 | 5.2 |
| 10 | 9 | AEBP1_HUMAN | Adipocyte enhancer-binding protein 1 | 0.007 | 0.039 | 5.2 |
| 2 | 1 | MOB1A_HUMAN | MOB kinase activator 1A | 0.000 | 0.002 | 5.2 |
| 3 | 1 | CSRP2_HUMAN | Cysteine and glycine-rich protein 2 | 0.000 | 0.001 | 4.8 |
| 3 | 2 | GRB2_HUMAN | Growth factor receptor-bound protein 2 | 0.001 | 0.003 | 4.8 |
| 2 | 1 | KAD3_HUMAN | GTP:AMP phosphotransferase AK3, mitochondrial {ECO:0000255\|HAMAP-Rule:MF_03169} | 0.000 | 0.001 | 4.7 |
| 4 | 2 | SSBP_HUMAN | Single-stranded DNA-binding protein, mitochondrial | 0.000 | 0.002 | 4.5 |
| 2 | 1 | IF1AX_HUMAN | Eukaryotic translation initiation factor 1A, X-chromosomal | 0.000 | 0.000 | 4.5 |
| 1 | 1 | PTPRK_HUMAN | Receptor-type tyrosine-protein phosphatase kappa | 0.000 | 0.002 | 4.5 |
| 2 | 1 | SVEP1_HUMAN | Sushi, von Willebrand factor type A, EGF and pentraxin domain-containing protein 1 | 0.000 | 0.001 | 4.4 |
| 1 | 1 | GSLG1_HUMAN | Golgi apparatus protein 1 | 0.000 | 0.001 | 4.3 |
| 8 | 3 | SYWC_HUMAN | Tryptophan--tRNA ligase, cytoplasmic | 0.001 | 0.006 | 4.2 |
| 2 | 2 | SPTB2_HUMAN | Spectrin beta chain, non-erythrocytic 1 | 0.001 | 0.002 | 4.1 |
| 2 | 1 | PRIO_HUMAN | Major prion protein | 0.001 | 0.003 | 4.1 |
| 6 | 1 | UB2L3_HUMAN | Ubiquitin-conjugating enzyme E2 L3 | 0.000 | 0.001 | 4.0 |
| 1 | 1 | H31T_HUMAN | Histone H3.1t | 0.001 | 0.002 | 4.0 |
| 5 | 2 | RNAS4_HUMAN | Ribonuclease 4 | 0.001 | 0.005 | 3.9 |
| 2 | 1 | AHSA1_HUMAN | Activator of 90 kDa heat shock protein ATPase homolog 1 | 0.001 | 0.003 | 3.8 |
| 2 | 1 | EIF3G_HUMAN | Eukaryotic translation initiation factor 3 subunit G {ECO:0000255\|HAMAP-Rule:MF_03006} | 0.000 | 0.001 | 3.7 |
| 8 | 3 | RACK1_HUMAN | Receptor of activated protein C kinase 1 | 0.003 | 0.009 | 3.4 |
| 1 | 1 | DUT_HUMAN | Deoxyuridine 5'-triphosphate nucleotidohydrolase, mitochondrial | 0.001 | 0.002 | 3.3 |
| 3 | 1 | ANT3_HUMAN | Antithrombin-III | 0.002 | 0.006 | 3.1 |
| 7 | 5 | DHE3_HUMAN | Glutamate dehydrogenase 1, mitochondrial | 0.007 | 0.021 | 3.1 |
| 1 | 1 | PROS_HUMAN | Vitamin K-dependent protein S | 0.000 | 0.001 | 3.0 |
| 1 | 1 | NB5R2_HUMAN | NADH-cytochrome b5 reductase 2 | 0.000 | 0.001 | 3.0 |
| 1 | 1 | HDGF_HUMAN | Hepatoma-derived growth factor | 0.001 | 0.002 | 3.0 |
| 1 | 1 | PIN4_HUMAN | Peptidyl-prolyl cis-trans isomerase NIMA-interacting 4 | 0.000 | 0.001 | 2.9 |
| 3 | 1 | TRFL_HUMAN | Lactotransferrin | 0.010 | 0.028 | 2.9 |
| 3 | 1 | PPIL1_HUMAN | Peptidyl-prolyl cis-trans isomerase-like 1 | 0.001 | 0.003 | 2.8 |
| 2 | 1 | HBA_HUMAN | Hemoglobin subunit alpha | 0.002 | 0.005 | 2.8 |
| 3 | 2 | RBM3_HUMAN | RNA-binding protein 3 | 0.001 | 0.004 | 2.7 |
| 1 | 1 | MSRA_HUMAN | Mitochondrial peptide methionine sulfoxide reductase | 0.000 | 0.001 | 2.6 |
| 3 | 3 | FETUA_HUMAN | Alpha-2-HS-glycoprotein | 0.007 | 0.017 | 2.3 |
| 4 | 4 | TRHDE_HUMAN | Thyrotropin-releasing hormone-degrading ectoenzyme | 0.004 | 0.008 | 2.3 |
| 2 | 1 | VTDB_HUMAN | Vitamin D-binding protein | 0.002 | 0.003 | 2.2 |
| 3 | 2 | SYNC_HUMAN | Asparagine--tRNA ligase, cytoplasmic | 0.001 | 0.002 | 2.2 |
| 3 | 2 | FBLN3_HUMAN | EGF-containing fibulin-like extracellular matrix protein 1 | 0.002 | 0.003 | 2.0 |
| 18 | 11 | HPLN1_HUMAN | Hyaluronan and proteoglycan link protein 1 | 0.072 | 0.142 | 2.0 |
| 7 | 3 | A2MG_HUMAN | Alpha-2-macroglobulin | 0.015 | 0.028 | 1.9 |
| 9 | 7 | SYHC_HUMAN | Histidine--tRNA ligase, cytoplasmic | 0.011 | 0.020 | 1.8 |
| 1 | 1 | RUXG_HUMAN | Small nuclear ribonucleoprotein G | 0.001 | 0.001 | 1.7 |
| 3 | 1 | SCOT1_HUMAN | Succinyl-CoA:3-ketoacid coenzyme A transferase 1, mitochondrial | 0.000 | 0.001 | 1.7 |
| 4 | 1 | TRFE_HUMAN | Serotransferrin | 0.000 | 0.001 | 1.6 |
