# Supplementary figures and images for: Impact of mesenchymal stem cells’ secretome on glioblastoma pathophysiology
Source: J Transl Med. 2017 Oct 2;15:200. doi: 10.1186/s12967-017-1303-8 (PMC5625623; doi:10.1186/s12967-017-1303-8)

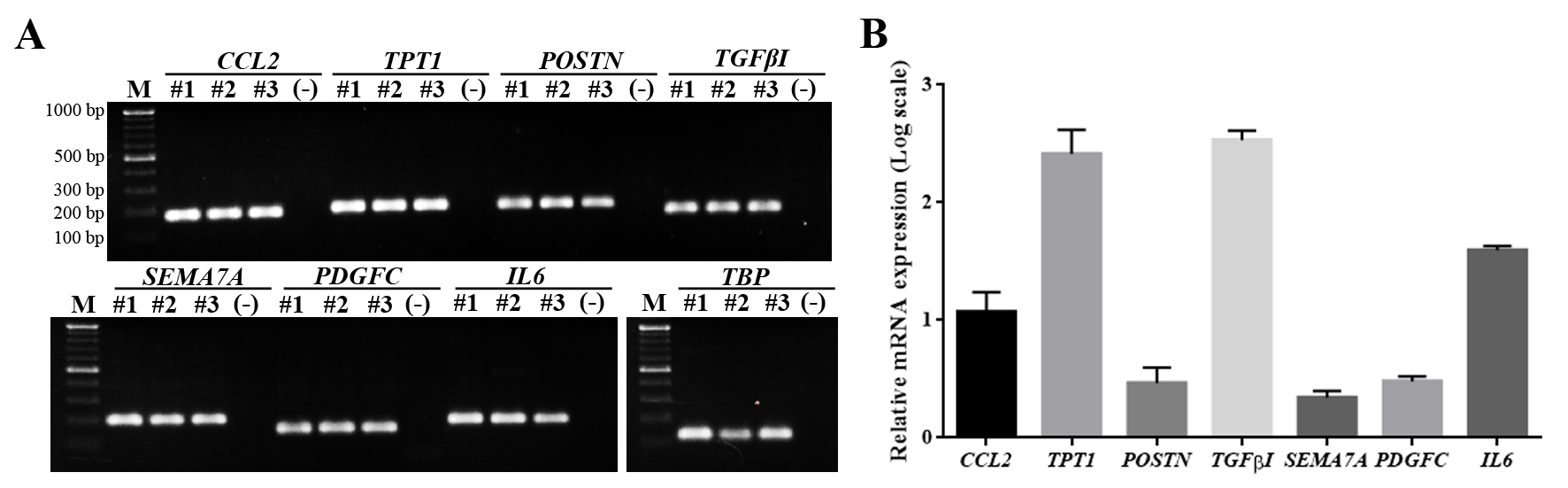

Supplement: Supplementary file 4 — Additional file 4: Figure S1. mRNA expression of genes coding for proteins detected in proteomic analyses by quantitative RT-qPCR. (A) Gel electrophoresis of CCL2, TPT1, POSTN, TGFβI, SEMA7A, PDGFC, IL6 and TBP expression in HUCPVCs. The RT-qPCR products were run on a 2% agarose gel. (B) Relative mRNA expression quantification in HUCPVCs. Data is normalized for TBP expression, and results are expressed as the mean ± SD of 3 biological replicates. M, Molecular weight marker 100 bp, ThermoScientific; #1, #2, and #3, independent biological replicates of HUCPVCs; (-), negative control. [file 12967_2017_1303_MOESM4_ESM.tif]
